# Measurement properties of the project-level Women's Empowerment in Agriculture Index

**DOI:** 10.1016/j.worlddev.2019.104639

**Published:** 2019-12

**Authors:** Kathryn M. Yount, Yuk Fai Cheong, Lauren Maxwell, Jessica Heckert, Elena M. Martinez, Gregory Seymour

**Affiliations:** a Emory University, United States; b International Food Policy Research Institute, United States; c Tufts University, United States

**Keywords:** BCC, Behavioral Change Communication, BRB, Building Resilience in Burkina Faso, CCC, Category Characteristic Curve, CFA, confirmatory factor analysis, CI, confidence interval, DIF, differential item functioning, EFA, exploratory factor analysis, GAAP2, Gender, Agriculture, and Assets Project Phase 2, GPI, gender parity index, IPV, intimate partner violence, IRT, item response theory, NRM, nominal response models, 2PL, two-parameter logistic, RAI, Relative Autonomy Index, TRAIN, Targeting and Realigning Agriculture for Improved Nutrition, WEAI, Women’s Empowerment in Agriculture Index, Agricultural development, Item response theory, Measurement, Sustainable development goals, Women’s agency, Women’s empowerment

## Abstract

Women’s empowerment is a process that includes increases in intrinsic agency (power within); instrumental agency (power to); and collective agency (power with). We used baseline data from two studies—Targeting and Realigning Agriculture for Improved Nutrition (TRAIN) in Bangladesh and Building Resilience in Burkina Faso (BRB)—to assess the measurement properties of survey questions operationalizing selected dimensions of intrinsic, instrumental, and collective agency in the project-level Women’s Empowerment in Agricultural Index (pro-WEAI). We applied unidimensional item-response models to question (item) sets to assess their measurement properties, and when possible, their cross-context measurement equivalence—a requirement of measures designed for cross-group comparisons. For *intrinsic agency in the right to bodily integrity*, measured with five attitudinal questions about intimate partner violence (IPV) against women, model assumptions of unidimensionality and local independence were met. Four items showed good model fit and measurement equivalence across TRAIN and BRB. For item sets designed to capture *autonomy in income*, *intrinsic agency in livelihoods activities*, and *instrumental agency in: livelihoods activities*, *the sale or use of outputs, the use of income*, and *borrowing from financial services*, model assumptions were not met, model fit was poor, and items generally were weakly related to the latent (unobserved) agency construct. For *intrinsic* and *instrumental agency in livelihoods activities* and for *instrumental agency in the sale or use of outputs and in the use of income,* items sets had similar precision along the latent-agency continuum, suggesting that similar item sets could be dropped without a loss of precision. IRT models for collective agency were not estimable because of low reported presence and membership in community groups. This analysis demonstrates the use of IRT methods to assess the measurement properties of item sets in pro-WEAI, and empowerment scales generally. Findings suggest that a shorter version of pro-WEAI can be developed that will improve its measurement properties. We recommend revisions to the pro-WEAI questionnaire and call for new measures of women’s collective agency.

## Highlights

• We tested the measurement properties of the project-level Women’s Empowerment in Agriculture Index in two projects/sites.
• We used IRT methods to assess item sets used in pro-WEAI indicators for intrinsic, instrumental, and collective agency.
• One item set capturing *intrinsic agency in the right to bodily integrity* was measurement equivalent across the two projects.
• We recommend refining the other item sets to improve their measurement properties.
• We offer steps to test, refine, validate, and shorten pro-WEAI and other women’s empowerment scales in development studies.

## 1 Introduction

2030 Sustainable Development Goal 5 (SDG5) prioritizes women’s empowerment and gender equality (United Nations General Assembly, 2015) in their own right and as drivers of other SDGs (UN Women, 2018). This mandate has mobilized efforts to conceptualize and to validate measures of women’s empowerment across population groups (Yount et al., nd, Yount et al., 2016), countries (Miedema, Haardörfer, Girard, & Yount, 2018), and time (Cheong, Yount, & Crandall, 2017). Findings from these studies show that selected measures of enabling resources and agency are comparable across social groups, contexts, and time, confirming the capacity to monitor SDG5 globally.

Central to this global monitoring effort has been the development and elaboration of the Women’s Empowerment in Agricultural Index (WEAI) (Alkire et al., 2013). Unlike other global measures of women’s empowerment, which are based on aggregate national data or country characteristics (United Nations Development Programme [UNDP], 2018, World Economic Forum, 2018), WEAI measures women’s empowerment in agriculture directly through household surveys of men and women and is based on a methodology for index construction designed originally to measure multidimensional poverty (Alkire & Foster, 2011).

Pro-WEAI, the latest adaptation of WEAI, is being developed as part of the Gender, Agriculture, and Assets Project Phase 2 (GAAP2).^1^ GAAP2, led by the International Food Policy Research Institute (IFPRI), includes 13 agricultural development projects in nine countries in South Asia and Sub-Saharan Africa that are piloting the pro-WEAI protocols. Pro-WEAI is designed for impact evaluations of agricultural development projects and includes new indicators, such as freedom of movement and attitudes about intimate partner violence (IPV) against women. In its aggregate, pro-WEAI provides an index of women’s empowerment designed for comparison across all groups, such as intervention arms, for which the dataset is representative. Pro-WEAI can be disaggregated into two sub-indices and 12 complementary indicators. Thus, change in the overall index value can be linked to changes in the joint distributions of sub-index and indicator-level achievements.

Given the need for valid measures of women’s empowerment to monitor SDG5 and design advantages of pro-WEAI, an assessment of its measurement properties is warranted. This paper has three aims: 1) to assess in two GAAP2 projects the measurement properties of survey question (item) sets used to compute pro-WEAI indicators, 2) to offer guidance, based on study findings, for questionnaire revisions to shorten the full pro-WEAI to improve it as a measure for women’s empowerment in agricultural development programs, and 3) to make a call for a validated ‘short form’^1^ version of pro-WEAI and improved measures of women’s collective agency.

## 2 Background

### 2.1 Framework for Women’s Empowerment

**Fig. 1:**
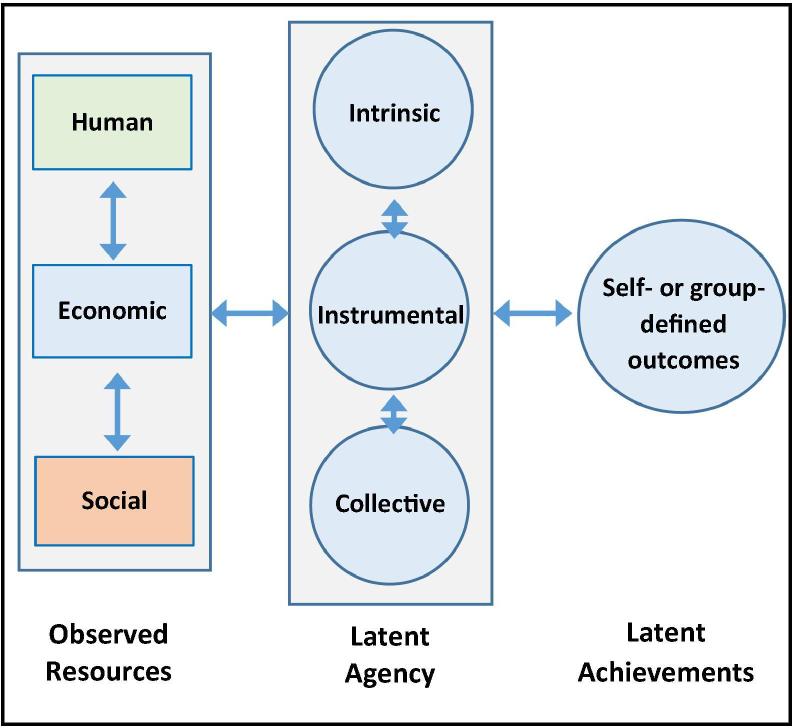
Framework for Women’s Empowerment.

*Women’s empowerment*, a multidimensional construct (Agarwala and Lynch, 2006, Lukes, 1974, Malhotra and Schuler, 2005, Mason, 2005), is the process whereby women claim new resources that may enhance their agency, or ability to make strategic life choices that enable them to achieve individual or collective goals (Kabeer, 1999). Human resources may include formal or informal schooling or training that expands valued knowledge or skills. Economic resources may include income, savings, or property. Social resources may include informal or formal networks of access or support, typically outside the family. We conceptualize resources as primarily observable, or measured directly in surveys, such as grades of schooling completed, chickens owned, or organizational memberships. Observed resource variables are depicted with squares in Fig. 1.

Agency is the ability to make strategic life choices in contexts where these choices once were denied (Kabeer, 1999). Contexts of constraint may include patriarchal family systems and institutions that privilege men, often the focus in discussions of women’s empowerment. Contexts of constraint also may include other oppressive systems, such as poverty. Pro-WEAI, and the framework presented here, conceptualizes agency as a multidimensional construct. Intrinsic agency—or power within—is the process by which one develops a critical consciousness of one’s own aspirations, capabilities, and rights (Batliwala, 1994, Freire, 1972, Kabeer, 1999, Komter, 1989, Stromquist, 1995). Instrumental agency—or power to—is strategic action to achieve one’s self-defined goals. Collective agency—or power with—is joint action to achieve shared goals (Bandura, 2000, Freire, 1972, Kabeer, 1999, Lukes, 1974, Rowlands, 1995, Rowlands, 1997, Rowlands, 1998, Stromquist, 1995). These types of agency are derived conceptually from multi-dimensional typologies of power described first by Komter (1989) with respect to gender and rooted in the seminal works of Freire, 1972, Lukes, 1974, who wrote on power and freedom from oppression without explicit reference to gender. ‘Power over,’ also discussed in this literature, is excluded from this framework and pro-WEAI, as it describes domination of one person or group over another (Weber, 1946). The idea of domination over others contradicts Kabeer’s definition and feminist cooperative ideas about power (Bologh, 2009). Achievements are the realizations of self- or group-defined goals, including outcomes related to personal and group well-being. The dimensions of agency and achievements^2^ are conceptualized here as latent constructs. A latent construct is not directly observed, and typically is measured with multiple, directly observed items, such as responses to a set of survey questions that together are expected to measure the latent construct. A woman’s unobserved status on each latent construct, or trait, is conceptualized to be the cause of her responses to the measured (or observed) items. Latent constructs (or traits) for agency and well-being achievements are depicted with circles in Fig. 1.

Although Kabeer defines resources as ‘pre-conditions’ for agency and the realization of life goals (Kabeer, 1999), she and others recognize that resources and agency are reciprocally related over time (Freire, 2018, Kabeer, 2005). As such, new claims on resources may enhance agency, which in turn, may foster new claims on other resources—individually and collectively. Thus, our framework (Fig. 1), and prior research (Yount, Crandall, & Cheong, 2018), recognize the reciprocal influences of dimensions of women’s empowerment over time.

### 2.2 Prior efforts to measure women’s empowerment and their limitations

Given the multidimensional, dynamic nature of women’s empowerment, prior efforts to operationalize and validate its dimensions have faced challenges. Global measures, like the Gender Gap Index, Gender Development Index, Gender Inequality Index, and Gender Empowerment Measure (World Economic Forum, 2018) either fail to measure women’s empowerment fully or rely on macro-level, proxy measures (Alkire, 2005, Alsop et al., 2006, Bardhan and Klasen, 1999, Charmes and Wieringa, 2003, Dijkstra, 2002, Dijkstra and Hanmer, 2000, Kishor and Subaiya, 2008, Klasen, 2006), such as life expectancy at birth, per capita income, schooling, or share of parliamentary seats. Such proxies are limited in their sensitivity to how gendered power relations govern women’s and men’s behavior at the micro level (Presser & Sen, 2000).

Direct measures of women’s empowerment in households and communities also have limitations. First, scholars have given more attention to measuring economic resources than to measuring human and social resources for women’s empowerment (Grootaert, Narayan, Jones, & Woolcock, 2004). Second, scholars have focused more on measuring instrumental agency than on measuring intrinsic and collective agency (James-Hawkins et al., 2016, Smith, 2003, Thorpe et al., 2015), such that transformative changes in intrapersonal critical consciousness and collective actions among women have been understudied (Brody et al., 2017, O’Hara and Clement, 2018). Third, scholars have tended to construct summative (or equally weighted) scales of agency constructs (Kumar et al., 2019, Mahmud et al., 2012), which ignores variation in the relationships of observed items with latent-agency constructs and possible systematic measurement error in these items. Fourth, with some exceptions (Agarwala and Lynch, 2006, Cheong et al., 2017, Crandall et al., 2015, Miedema et al., 2018, Yount et al., nd, Yount et al., 2014, Yount et al., 2016), scholars have not fully assessed the measurement properties of agency scales, including their measurement equivalence across meaningful groups, such as program beneficiaries and non-beneficiaries, program types, geographic contexts, and time. Consequently, the ‘end users’ of tools to measure women’s empowerment cannot discern the utility of one scale over another, and researchers and practitioners continue to construct measures using inconsistent terms, item sets, and methods, diminishing the capacity to make meaningful global comparisons.

Novel approaches to validate measures of women’s intrinsic agency and instrumental agency in the household/family have included the use of psychometric methods, such as factor analysis, item response theory (IRT) methods, and structural equation modeling (Cheong et al., 2017, Crandall et al., 2015, Miedema et al., 2018, Yount et al., 2014, Yount et al., 2016). Such methods help to identify survey-question sets that are valid, observed items of latent constructs, like women’s agency. To be valid, item sets should operationalize well-defined constructs and should be empirically (or psychometrically) ‘comparable’ across settings, social groups, and time. Using these methods, Yount and colleagues have identified three indices of women’s intrinsic agency. The first—women’s expressed right to bodily integrity—uses nine attitudinal questions about IPV against wives, derived from the those used across the Demographic and Health Surveys (ICF International, 2011) and some of which are included in pro-WEAI (Malapit et al., 2019). These items are psychometrically comparable across genders (Yount, VanderEnde, Zureick-Brown, Hoang, et al., 2014), age-at-marriage groups (Yount et al., 2016), and countries (Miedema et al., 2018). The second index—women’s expressed self-efficacy—uses items from the general self-efficacy scale (Scholz et al., 2002, Sherer et al., 1982), which overlap with those used in the pro-WEAI (Malapit et al., 2019). The items are validated in young Qatari women (Crandall et al., 2015). The third index—women’s expressed social and economic rights—uses attitudinal items not in the pro-WEAI but derived from qualitative research and psychometrically comparable across Qatari and non-Qatari women (Yount et al., nd).

Using these methods, Yount and colleagues also have identified two indices for women’s instrumental agency in the household/family. The first index—women’s influence in household/family decisions—uses items capturing decisions about a woman’s earnings, her husband’s earnings, large household purchases, daily household purchases, seeking medical treatment, and visits to family and friends; thus, some of these items overlap with those in the pro-WEAI. These items that are validated at the national level (Miedema et al., 2018, Yount et al., 2016) as well as across age-at-marriage groups (Yount et al., 2016), multiple East African countries (Miedema et al., 2018), and time (Cheong et al., 2017). The second index—women’s freedom of movement—captures their ability to visit venues outside the home uses three items distinct from those in the pro-WEAI but validated at the national level (Yount et al., 2016) and across age-at-marriage groups (Yount et al., 2016) and time (Cheong et al., 2017). These efforts have identified general measures of women’s intrinsic agency and instrumental agency in the household/family that are empirically comparable across diverse contexts, population groups, and time periods. The successful validation of measures of intrinsic agency and instrumental agency in the household/family invoke a call to validate similar measures for women’s agency in other sectors, such as in agriculture (Beghini, Cattaneo, & Pozzan, 2019).

### 2.3 Pro-WEAI as a measure of women’s empowerment in agricultural development programs

#### 2.3.1 The Women’s Empowerment in Agriculture index (WEAI)

In 2012, the US Agency for International Development, IFPRI, and the Oxford Poverty and Human Development Initiative launched the Women’s Empowerment in Agriculture Index (WEAI) (Alkire et al., 2013) as a monitoring and evaluation tool to compare population levels and changes over time in women’s empowerment in agriculture across countries, regions, and population groups. The WEAI measures women’s empowerment through a household survey that focuses conceptually on women’s agency. The WEAI consists of two sub-indices. The Five Domains of Empowerment Index (5DE) is designed to measure the incidence (or headcount) and intensity of dis-empowerment. The Gender Parity Index (GPI) is designed to provide information on women’s empowerment relative to that of men in their households (Alkire et al., 2013).

#### 2.3.2 Purpose of the pro-WEAI

Pro-WEAI, the most recent adaptation of WEAI, is designed to diagnose disempowerment and to assess the impact of agricultural development projects on empowerment. The WEAI and pro-WEAI are based on the Alkire-Foster counting methodology for index construction (Alkire and Foster, 2011, Malapit et al., 2019), applied to measure intrinsic, instrumental, and collective agency; however, the two indices differ in notable ways. First, the WEAI is designed to capture national levels and trends in women’s empowerment in agriculture; whereas, pro-WEAI is designed for impact evaluations of agricultural development projects. Second, pro-WEAI includes new indicators, such as IPV attitudes and freedom of movement.

Pro-WEAI provides an ‘information platform’ (Alkire, 2018) to measure women’s empowerment in agriculture and, to some extent, more broadly. As a headline figure, pro-WEAI provides an overall measure of women’s empowerment in agriculture that is designed to be comparable at all levels for which the data are representative, such as intervention or population groups. Pro-WEAI also can be disaggregated into two sub-indices—the 3DE (like the 5DE, but refers to intrinsic, instrumental, and collective agency) and GPI—and into 12 indicators, each of which is designed to capture distinct aspects of intrinsic, instrumental, or collective agency (Malapit et al., 2019). Pro-WEAI indicators for intrinsic agency include autonomy in income, self-efficacy, attitudes about IPV, and respect among household members. Pro-WEAI indicators for instrumental agency include input into productive decisions, ownership of assets, access to and decisions on credit, control over use of income, work balance, and visiting important locations. Pro-WEAI indicators for collective agency include group membership and membership in influential groups. Using this decomposition, one can, in theory, assess how changes in the joint distribution of indicator-level achievements contribute to changes in the overall index value. The capacity for this decomposition stems from the counting-based approach used to construct pro-WEAI, which requires that the definitions, thresholds, and weights used to create each indicator are explicit.

Some may argue that the broad applicability of pro-WEAI may impede the accurate measurement of women’s empowerment in agriculture in local contexts. Indeed, debate continues about the universality or context-specificity of measures for women’s empowerment (Alkire et al., 2013, Malhotra and Schuler, 2005, Mason, 1986, Mason and Smith, 2003, Richardson, 2018, Yount et al., nd). What may be indicative of empowerment among women in Bangladesh—such as, joint decision making on salient agricultural decisions—may not be indicative of empowerment among women in Ghana, where norms around agriculture differ (Seymour & Peterman, 2018). Although pro-WEAI was designed to be comparable across different agricultural systems, countries, and cultural contexts (Malapit et al., 2019), pro-WEAI does not ignore cultural differences. The design of pro-WEAI involved qualitative research to explore concepts of empowerment in diverse rural settings, and the suite of pro-WEAI instruments includes customizable qualitative guides designed to capture nuanced local meanings and processes of women’s empowerment (Meinzen-Dick, Rubin, Elias, Mulema, & Myers, 2019). Also, the survey-based pro-WEAI index has undergone sensitivity analysis to test its robustness to alternative specifications (Malapit et al., 2019). The conceptual basis for pro-WEAI, for example, which indicators to include and how to define and weight them, does not prioritize any one country or context over another. In practical terms, the indicators in pro-WEAI are defined and weighted to be applicable across the widest possible set of circumstances.

Given the design advantages of pro-WEAI, this analysis aimed to assess the measurement properties of survey question (item) sets in pro-WEAI related to intrinsic, instrumental, and collective agency and to make recommendations for pro-WEAI’s refinement as an impact evaluation tool. Extending prior work (Pitt, Khandker, & Cartwright, 2006), we leveraged item-response theory (IRT) methods to assess the measurement properties of the aforementioned item sets, collected across two GAAP2 projects in Bangladesh (South Asia) and Burkina Faso (West Africa). The analysis reveals the utility of IRT methods for assessing comparatively the measurement properties of item sets used to construct selected pro-WEAI indicators, guiding refinements of pro-WEAI to improve its indicator-specific and overall measurement properties, and underscoring the value of shortening the full pro-WEAI and creating from that a validated short-form pro-WEAI for national and program-level monitoring.

## 3 Methods

### 3.1 Study contexts

This analysis uses quantitative baseline data from two GAAP2 projects: Targeting and Realigning Agriculture to Improve Nutrition (TRAIN) in Bangladesh and Building Resilience in Vulnerable Communities in Burkina Faso (BRB). BRAC (Building Resources Across Communities) is implementing TRAIN, and IFPRI is evaluating it. The project aims to improve women’s and children’s nutrition by diversifying production and farmers’ incomes; educating participants about nutrition and health; increasing women’s control over credit; and sensitizing men on women’s role in agriculture and family care. The intervention package includes behavior change communication (BCC) on nutrition, health, and sanitation; an agricultural credit program targeted to women farmers; nutrition-sensitive agricultural extension targeted to men and women; and a component on men’s sensitization and social mobilization delivered through a community-based empowerment program. The project targets young women who are likely to become pregnant or to give birth in the near future in 144 geographical unions over four years. The evaluation design is a cluster-randomized controlled trial with four arms: 1) a comparison group receiving the agricultural credit program only and intervention groups receiving 2) agricultural credit with BCC, 3) agricultural credit with BCC and agricultural extension, and 4) agricultural credit with BCC, agricultural extension, and men’s sensitization/community mobilization.

The Grameen Foundation is implementing BRB, and the evaluation is in partnership with a researcher at Brigham Young University. BRB aims to improve household income and nutrition and to empower women by building and supporting community-based women’s savings groups; educating participants about agricultural business and nutrition; facilitating dialogues on gender roles in agriculture and diets; and linking participants to agricultural services and financing. BRB is targeting 80,000 women in rural areas of Central-Western Burkina Faso over three years. The evaluation has a pre-test/post-test, quasi-experimental design, where the intervention group is women in savings groups who received the BRB intervention package, and the comparison group is women in similar savings groups in non-program areas who did not receive the BRB intervention package.

### 3.2 Samples and data

This analysis uses data from the baseline pro-WEAI survey from each project (Appendix 1). The baseline survey in TRAIN was administered between November 2016 and February 2017 to 5040 households in which at least one woman 18–35 years was present (some households did not include a male adult). The baseline survey in BRB was administered in May 2016 to a subset of households, including 380 women (190 intervention group; 190 comparison group), as well as their husbands or the male heads of household, for a total of 760 respondents. Only the women’s responses were analyzed here.

The topics covered in the pro-WEAI survey build on prior work developing the WEAI. Additional topics to include were identified based on consultations with implementers of the gender-sensitive agricultural development programs in the GAAP2 portfolio and other gender experts during the project inception workshop in January 2016 (Malapit et al., 2019). The target beneficiary and spouse were asked questions about household decision-making around production and use of income; access to productive capital; access to financial services; time allocation; group membership; frequency and freedom of movement; intra-household relationships including respect for household members; autonomy in decision-making using vignettes inspired by the Relative Autonomy Index (RAI) (Ryan & Deci, 2000); self-efficacy using the new general self-efficacy scale (Chen, Gully, & Eden, 2001); and attitudes about IPV against women using validated items from the Demographic and Health Surveys (DHS) (Yount, VanderEnde, Zureick-Brown, Hoang, et al., 2014).^3^ Furthermore, qualitative work tied to the projects in the GAAP2 portfolio has identified these topics as ones that are important to the study population (Meinzen-Dick et al., 2019).

Data for descriptive analysis of the two samples consisted of socioeconomic and demographic information from the household rosters. Data that were considered for the IRT analyses consisted of responses to eight question (item) sets that were collected in both project sites and that captured four dimensions of intrinsic agency and four dimensions of instrumental agency. Our conceptual organization of item sets in the present analysis differed somewhat from the 12 pro-WEAI indicators because we mapped the wording of pro-WEAI questions to the definitions of intrinsic agency (as critical consciousness of capabilities and rights) and instrumental agency (as behavioral action). Appendix 2 compares the item sets as we have organized them with their contribution to each pro-WEAI indicator (Malapit et al., 2019). Appendix 2 also clarifies the rationale for not including the remaining item sets from one or the other project in this comparative analysis.

Here, *intrinsic agency in the right to bodily integrity* was captured using women’s yes/no responses to the question, ‘Is a husband justified in hitting his wife if…,’ in five situations, such as ‘she burns the food?,’ ‘she goes out without telling him?,’ and ‘she neglects the children?’^4^ In East Africa, these items were validated as a unidimensional construct and were correlated with other agency constructs (Miedema et al., 2018). Intrinsic agency or *autonomy in use of income* was captured using vignettes, inspired by the Relative Autonomy Index (Ryan & Deci, 2000), which sought to measure the motivations behind women’s actions with respect to their income, distinguishing external and internal forms of regulation. We analyzed women’s responses to the question, ‘How similar are you to someone who…’ behaves in four ways with respect to her income, including ‘uses her income as determined by necessity,’ ‘uses her income how her family or community tells her she must’ (external), ‘uses her income how her family or community expects because she wants them to approve of her’ (external) and ‘chooses to use her income how she wants to and thinks is best for herself and her family’ (internal).^5^ Ordinal response options were completely the same (=0), somewhat the same (=1), somewhat different (=2), and completely different (=3). *Intrinsic agency in livelihoods activities* was captured using women’s responses to the question, ‘To what extent do you feel you can participate in decisions regarding [ACTIVITY] if you want(ed) to.’ Of 10 activities listed, examples were ‘raising poultry,’ ‘high-value crop farming,’ and ‘wage or salary employment.’ Response options captured participation (yes/no) and the extent that participants felt able to influence decisions about the activity (0 = not at all, 1 = small extent, 2 = medium extent, 3 = large extent). *Intrinsic agency in group membership* was captured using women’s responses to the question, ‘To what extent do you feel you can influence decisions in [GROUP]?’^6^ Examples of eight groups listed were ‘agriculture/livestock,’ ‘credit or microfinance,’ and ‘religious.’ Response options captured the presence of a group (yes/no), active membership in the group (yes/no), and the extent that active members felt they could influence group decisions (0 = not at all, 1 = small extent, 2 = medium extent, 3 = high extent).

*Instrumental agency in livelihoods activities* was captured using women’s responses for the same 10 activities (as above) to the question, ‘How much input did you have in making decisions about [ACTIVITY].’ *Instrumental agency in the sale or use of outputs* from 6 of the 10 (agricultural) livelihoods activities was captured using women’s responses to the question, ‘How much input did you have in decisions about…how much of the outputs of [ACTIVITY] to keep for consumption at home rather than selling?’ *Instrumental agency in the use of income* generated from 8 of the 10 livelihoods activities was captured using women’s responses to the question, ‘How much input did you have in decisions about…how to use income generated from [ACTIVITY].’ Response options for all 10 livelihoods activities were partially ordered by design, in that a nominal category captured women’s non-participation in each activity, and ordered categories captured the amount of input that *participants* reported having in decisions about each activity, its outputs, or income generated (0 = little/no decisions, 1 = some decisions, 2 = most/all decisions).^7^ Finally, *instrumental agency in borrowing from financial services* was captured using women’s responses to three questions, ‘Who made the decision to borrow from [SOURCE] most of the time?’, ‘Who made the decision about what to do with the money from [SOURCE] most of the time?’ and ‘Who was responsible for repaying the money borrowed from [SOURCE]?’ Examples of the six financial services listed were specific formal lenders, ‘informal lender,’ and ‘friends or relatives,’ and response options were nominal, capturing first whether the household was able to borrow from each source if it wanted to (yes/no), then whether the household borrowed from this source in the past 12 months (yes/no), and if so, whether the respondent was involved in decisions about borrowing (yes/no).^8^

Accounting for skip patterns in the pro-WEAI questionnaire, item-level missingness due to non-response was generally low across project sites and constructs of agency (Results). For all IRT analyses except one, missingness was included as a response category, so the influence of missingness on estimated model parameters could be assessed. In the IRT analysis of *instrumental agency in income*, only one observation was missing data on staple grain farming, so missingness could not be included as a response category. This observation was dropped from the analysis.

### 3.3 Analysis

We chose item response theory (IRT) methods to examine item sets designed to measure each dimension of agency in pro-WEAI in the two project sites that met the requisite assumptions. IRT methods, a family of statistical techniques for analyzing latent variables, allow researchers to assess the empirical relationships between observed items, such as responses to survey questions, that are theorized to be causal expressions of a person’s status along the continuum of an unobserved (latent) trait (Lord, 1980). IRT methods have several advantages over other psychometric methods in scale development. First, IRT methods uniquely allow comparison of estimated latent traits and item characteristics, as they are placed on a common scale. Second, IRT methods allow estimation of the standard error of measurement, which may differ across levels of the latent trait and is general across populations (Embretson & Reise, 2000). Third, IRT methods allow items to vary in difficulty, and take this information into account when scaling the items. Fourth, IRT methods are useful to explore and to test the functional form of item-level response options, such as those intended to be ordinal (e.g., 0 = not at all, 1 = small extent, 2 = medium extent, 3 = high extent). Third, IRT methods can be applied to reduce a valid ‘long form’ (full pro-WEAI) to a valid short-form (short-form pro-WEAI) (Meade & Lautenschlager, 2004) that captures, as precisely as possible, the desired range of values along each latent-trait continuum being measured.^9^

Here, we followed the steps described in Toland (2014) and in Tay, Meade, and Cao (2015) to assess the measurement properties of item sets designed to measure dimensions of agency collected in the baseline pro-WEAI surveys of two independent GAAP2 projects. We assessed the item sets, described above, designed to measure *intrinsic agency in*: *the right to bodily integrity*, *autonomy in use of income*, *livelihoods activities*, and *group membership*. We also assessed the item sets, described above, designed to measure *instrumental agency in*: *livelihoods activities*, *the sale or use of outputs generated from livelihoods activities*, *the use of income generated from livelihoods activities*, and *borrowing from financial services*. Together, these item sets contributed to six of the 12 pro-WEAI indicators: autonomy in income, attitudes about IPV against women, input in productive decisions, access to and decisions on financial services, control over use of income, and group membership (Malapit et al., 2019). Our analytic steps are summarized in Table 1.

**Table 1:** General Steps in Item Response Theory (IRT) Analysis of Measurement Properties of Women’s Empowerment Scales.

Step	Description	Procedures for Analysis
1	Clarify Purpose of Study	Assess the measurement properties of item sets used to construct pro-WEAI indicators before using the indicators and overall index for impact evaluation of GAAP2 projects. The analysis is designed to ensure that item-sets assessed are as precise as possible across a desired score range or suitably matched to latent trait levels of the intended population. Key questions addressed: 1. Is the nature of the response set (binary, ordinal) stable across the response category system? 2. What is the level of measurement precision across the agency continua? 3. Are there redundant items that can be dropped? 4. Are there any gaps on the measured continua?
2	Consider Relevant Models	1. Items sets with binary response options: 2 parameter logistic (2PL) or 1 parameter logistic (1PL) IRT models 2. Example: Attitudes about IPV against women 3. Item sets with ordered/Likert-type response options: Graded IRT model 4. Example: Autonomy in income 5. Item sets with a partially ordered response options: nominal IRT model 6. Example Intrinsic agency in livelihoods activities
3	Conduct Preliminary Data Inspection	1. Are there adequate numbers of observations in each response category per item? 2. Should response options with few observations be collapsed?
4	Evaluate Model Assumptions and Test Competing Models	1. Dimensionality (in our case, unidimensionality) before IRT estimation using exploratory factor analysis (EFA) or confirmatory factor analysis (CFA) depending upon the stage of development and prior validation of the scales 2. Local independence (LI) within items sets using standardized LD χ2 statistic for item pairs LD χ2 < |5| likely local independence LD χ2 > |5| questionable LD LD χ2 > |10| likely LD Note: If assumptions 1 and 2 are not met, IRT model parameter estimates are not presented, as the parameter estimates and scores may be distorted 3. Functional form of response options using visual or graphical inspection a. Assess model-data fit at item-level using standardized X^2^ statistic at item-level b. Assess model-data fit at model-level by comparing BIC (Bayesian information criterion) and AIC (Akaike information criterion)—both relative information criteria—of base and competing model; smaller values for BIC and AIC indicate better model fit c. Assess functional form of response options with graphical displays 4. Normality of distribution of latent variable in the population (assumed with use of IRT methods)
5	Evaluate and Interpret Results	Assess item properties with item characteristic curves (ICCs) and item information curves (IICs)Assess scale properties with total information functions (TIFs)Produce IRT score estimates
6	Perform Measurement Equivalence Analysis	1. Assess measurement equivalence of item sets across projects/social groups (in our case TRAIN and BRB) 2. Estimate the effect size of the differential item functioning, if detected

Note. Adapted from Toland, 2014, Tay et al., 2015.

After clarifying the purpose of the analysis (Step 1), we considered relevant item response models for each item set (Step 2). We estimated unidimensional IRT models for item sets theorized to capture single indicators for agency. For the five IPV-attitudes items capturing women’s *intrinsic agency in the right to bodily integrity*, we chose a two-parameter logistic (2PL) response model for dichotomous outcomes, expressed as:

(1) $$Pr\left(X_{\mathrm{is}}=1|\theta_{s},b_{i},a_{i}\right)=\frac{exp[a_{i}\left(\theta_{s}-b_{i}\right)]}{1+exp[a_{i}\left(\theta_{s}-b_{i}\right)]}$$

where X_is_ denotes the response of woman s to item i (0 or 1), Θ_s_ denotes the ‘ability’ or level of the latent trait Θ for woman s, b_i_ denotes the threshold or ‘difficulty’ of item i, and a_i_ denotes the slope or ‘discrimination’ of item i. The difficulty refers to the level of the latent trait at which the probability of an endorsed response to the item—IPV *not justified* (=1)—is 0.5.^10^ The discrimination refers to an item’s capacity to distinguish respondents at specific levels of the latent-agency trait, with larger values suggesting greater discrimination.

For *autonomy in use of income*, we chose the graded-response (GR) model (Samejima, 1969), which can be considered an extension of the 2PL model for use with items having two or more ordered response categories k, where k = 1,…,K. For items designed to measure autonomy in income, response options were designed to be ordered on a 0–3 scale, as described above. The GR model estimates a unique discrimination parameter for each item *across* its K ordered response categories as well as K – 1 between-category thresholds, which indicate the level of the latent-agency trait needed to have a 50% chance of responding above one of the K-1 thresholds between the response categories.

Finally, for *intrinsic agency in livelihoods activities* and *instrumental agency in livelihoods activities*, *instrumental agency in the sale or use of outputs, instrumental agency in the use of income*, and *instrumental agency in borrowing from financial services*, respondents were asked about their participation (yes/no), and only participants were asked about their level of agency with respect to each activity, scored 0–2 or 0–3, as discussed, above. Although the response options for items in these sets appeared to be partially ordered, we used a nominal response model (NRM) (Bock, 1972) to test this assumption. Thus, NRMs are a class of IRT models that handle unordered, polytomous data.

After considering the relevant item response models, we conducted univariate analysis of the TRAIN and BRB samples to describe their demographic characteristics and to explore all item sets planned for inclusion in the IRT analyses (Step 3). This step helped to ensure that response options were not too sparse and the missing-at-random assumption was reasonable.

Then, before fitting IRT models, we assessed the assumption of unidimensionality for item sets with binary and ordered response options (IPV-attitudes and autonomy in income, respectively) (Step 4). This assumption—that one continuous latent variable can explain the item responses (Toland, 2014)—is implied in the construction of each pro-WEAI indicator. Unidimensionality can be assessed *a priori* using non-IRT methods, such as exploratory factor analysis (EFA) or confirmatory factor analysis (CFA) for dichotomous and ordered polytomous items. EFA is recommended when minimal prior research exists on a construct; whereas, CFA is recommended for well-theorized, validated constructs. Because prior WEAI instruments and theory informed the items sets used to construct the pro-WEAI indicators, we performed CFA separately for the five IPV-attitudes items and for the four autonomy-in-income items. We used three indices to assess fit for a unidimensional CFA model: the comparative fit index (CFI should be ≥0.95), Tucker Lewis Index (TLI should be ≥0.95), and root-mean-square error of approximation (RMSEA should be ≤0.06 and 90% CI ≤ 0.06) (Hu and Bentler, 1999, Yu, 2002). Because CFA is not well suited for item sets with nominal or partially ordered response options (Chen, Wang, & Chen, 2012), we skipped the step of testing unidimensionality for the other item sets.

Then, for agency item sets with well-fitting unidimensional CFA models, or with nominal response options, we performed IRT analyses, evaluating model assumptions and testing competing models (Step 4). We first evaluated the model assumption of local independence (LI). LI means that the latent trait variable being measured is the only influence on a woman’s response to an agency item. No other agency item and no other latent trait variable influences the woman’s item responses. Thus, for a given woman with a known agency score, her response to one item is independent of her response to any other item. Violating the LI assumption is problematic because model estimates, model fit statistics, and derived scores and associated standard errors can be distorted, and thus, differ from the construct being measured (Toland, 2014). LD can occur, for example, when similar wording is used across question stems or items, such that women cannot distinguish them and select the same response category repeatedly. To assess the LI assumption, we used the (approximately) standardized LD χ2 statistic for each item pair in a set (Chen & Thissen, 1997). LD χ2 statistics greater than |10| were considered large, providing evidence of probable LD and residual variance unaccounted for by the unidimensional IRT model. LD χ2 statistics between |5| and |10| were considered moderate, providing evidence of possible LD. LD χ2 statistics less than |5| were considered small and inconsequential (Cai et al., 2011, Cai et al., 2011). For item sets with no substantial violations observed in terms of the proportions of LD X2 statistics suggesting moderate and possible LD, sensitivity analyses were performed in which single items with the highest LD χ2 statistic were removed to assess their impact on violation of the LI assumption and to see if an item subset could be found that met the LI assumption.

Other assumptions assessed were functional form and model-data fit (Step 4). Regarding functional form, the GR model implies that all threshold parameters are ordered and the items share a common slope (Toland, 2014). To assess functional form, we graphed all response option functions against the latent-trait continuum to check whether each theoretically higher response option was more likely to be selected than prior response options at higher levels of the latent-trait continuum. We then assessed model-data fit at the item and model levels. The standardized chi-squared (S-Χ^2^) item-fit statistic was used to test the degree of similarity between model-predicted and empirical (observed) response frequencies for each item response category. A statistically significant S-Χ^2^ value indicated the model did not fit a given item. Poorly fitting items were candidates for removal, usually one at a time; and the item response model was re-estimated with the remaining items. With reasonable item-level model fit, we then assessed model-level fit by comparing IRT models with different levels of complexity.^11^ We used the Bayesian information criterion (BIC) and the Akaike information criterion (AIC) to compare the fits of competing models.

When model assumptions held, we then described, graphically and numerically, the item properties that included the estimates of the thresholds and slopes as well as the precision for each item, item subset, or full scale at a particular location or range of the latent-trait continuum. Item characteristic curves (ICCs) related the probability of endorsing each response option (e.g., 0 = IPV justified versus 1 = IPV not justified) for an item as a function of the level of the latent-agency trait. Together, the ICCs allowed us to assess visually the distribution of the location parameters for each item along the latent-trait continuum, the strength of the relationship between each item and the latent trait (discrimination), and if items had multiple response options, their empirical ordering. Item information curves (IICs) provided information about the precision of a specific item along the latent-trait continuum. The total information curves (TICs) depicted the sum of the IICs and indicated the precision of the entire item set along the latent-trait continuum. IICs and TICs could be used to decide which item pairs or sets had similar (redundant) precision, and therefore, were candidates for dropping.

Finally (Step 6), we assessed the measurement equivalence for item sets across the TRAIN and BRB samples that met IRT model assumptions of unidimensionality, local independence, and within-setting model-data fit. Following established guidelines (Tay et al., 2015), we investigated whether any items displayed differential item functioning by comparing the difficulty and discrimination parameters across TRAIN and BRB, holding constant the latent-trait level.

We used Stata SE version 15.1 (StataCorp, 2017) for descriptive analyses and data manipulation. We used Mplus (Muthén & Muthén, 1998-2017) to perform CFA to evaluate the assumption of unidimensionality for each set of IRT models. We used IRTPRO version 4.1 (Cai et al., 2011, Cai et al., 2011) for IRT analyses and Stata (StataCorp, 2017) to prepare graphs to summarize the results. Cheong, Maxwell, and Yount (nd) provide a guide for implementing IRT models in IRTPRO.

## 4 Results

### 4.1 Characteristics of respondents

As shown in Table 2, most women in the TRAIN sample had received some formal schooling.^12^ Few women in either sample participated in wage employment (9% TRAIN; 19% BRB). Among women who participated in non-agricultural activities, most reported being able to access the information they needed to make informed decisions. Qualitatively, relatively fewer women in TRAIN than in BRB solely or jointly cultivated land (24% versus 99%) and solely or jointly owned land (11% versus 66%). A minority of women in both samples (32% TRAIN, 11% BRB) solely or jointly held an account at a bank or other formal financial institution. Most women in both samples reported being able to access basic food, clothing, and medicines for themselves and their children. Among women who had children under five in both samples, most reported having access to child care, if needed. The ability of households to borrow money from various sources differed across samples. Qualitatively, a lower percentage of households in TRAIN than BRB reportedly could borrow from group-based microfinance (35% versus 76%) or informal credit groups (24% versus 96%); whereas a higher percentage of households in TRAIN than BRB reportedly could borrow from formal (64% versus 25%) and informal (62% versus 28%) lenders.

**Table 2:** Sample Characteristics, pro-WEAI Baseline Surveys, Women Participating in TRAIN Bangladesh and BRB Burkina Faso GAAP2 Projects.

	TRAIN, Bangladesh (N = 5040)								BRB, Burkina Faso (N = 380)							
	Yes		No						Yes		No					
**Human Resources**	N	(%)	N	(%)					N	(%)	N	(%)				
Any formal schooling^a^	4655	92.4	385	7.6												
To what extent are you able to access information you feel is important for making informed decisions about:	Medium or high extent		Not at all/small extent		Resp. doesn’t participate				Medium or high extent		Not at all/small extent		Resp. doesn’t participate			
Non-farm economic activities	472	(9.4)	147	(2.9)	4421	(87.7)			195	(51.3)	23	(6.1)	162	(42.6)		
Wage employment	304	(6.0)	140	(2.8)	4596	(91.2)			65	(17.1)	8	(2.1)	307	(80.8)		
Large household purchases	1106	(21.9)	586	(11.6)	3348	(66.4)			251	(66.1)	129	(40.0)				
Routine household purchases	3418	(67.8)	960	(19.1)	662	(13.1)			258	(67.9)	122	(32.1)				
**Economic Resources**	Yes		No		Missing				Yes		No		Missing			
Respondent solely or jointly cultivates land	1208	(24.0)	3832	(76.0)					376	(99.0)	1	(0.3)	3	(0.8)		
Respondent solely or jointly owns land cultivated by her household	539	(10.7)	4501	(89.3)					250	(65.8)	130	(34.2)				
Respondent solely or jointly holds financial account at bank or other formal institution	1586	(31.5)	3442	(68.3)	12	(0.2)			41	(10.8)	323	(85.0)	16	(4.2)		
If you needed to, could you acquire:	Yes		No		Not applicable				Yes		No		Not applicable		Missing	
Small amounts of food	4373	(86.8)	660	(13.1)	7	(0.1)			340	(89.5)	38	(10.0)	2	(0.5)		
Large amounts of food	4066	(80.7)	970	(19.3)	4	(0.1)			302	(79.5)	71	(18.7)	7	(1.8)		
Eggs	4532	(89.9)	498	(9.9)	10	(0.2)			322	(84.7)	32	(8.4)	26	(6.8)		
Milk	4494	(89.2)	536	(10.6)	10	(0.2)			334	(87.9)	29	(7.6)	17	(4.5)		
Meat/poultry/fish	4263	(84.6)	762	(15.1)	15	(0.3)			341	(89.7)	27	(7.1)	5	(1.3)	7	(1.8)
Special foods for children	3462	(68.7)	537	(10.7)	1.041	(20.7)			264	(69.5)	81	(21.3)	32	(8.4)	3	(0.8)
Nutritious foods recommended by healthcare worker	3431	(68.1)	714	(14.2)	895	(17.8)			266	(70.0)	87	(22.9)	23	(6.1)	4	(1.1)
Medication or vitamins for your children	3669	(72.8)	547	(10.9)	824	(16.4)			285	(75.0)	69	(18.2)	26	(6.8)		
Medication or vitamins for you	4285	(85.0)	705	(14.0)	50	(1.0)			289	(76.1)	71	(18.7)	20	(5.3)		
Clothing for your children	3778	(75.0)	529	(10.5)	733	(14.5)			362	(95.3)	10	(2.6)	8	(2.1)		
Clothing for you	4269	(84.7)	756	(15.0)	15	(0.3)			364	(95.8)	13	(3.4)	3	(0.8)		
Toiletries	4468	(88.7)	567	(11.3)	5	(0.1)			368	(96.8)	9	(2.4)	3	(0.8)		
**Social Resources**																
Has someone to watch child <5 so she can do things she needs to do	2756	(54.7)	238	(4.7)	2.046	(40.6)			148	(39.0)	17	(4.5)	202	(53.2)	13	(3.4)
**Household resources**																
Household owns or cultivates land	4998	(99.2)	42	(0.8)					377	(99.2)	3	(0.8)				
Household member could borrow cash/in kind from:	Yes		No		Maybe		Missing		Yes		No		Maybe			
NGO	4754	(94.3)	262	(5.2)	23	(0.5)	1	(<0.1)	174	(45.8)	192	(50.5)	14	(3.7)		
Formal lender (bank/financial institution)	3206	(63.6)	1618	(32.1)	215	(4.3)	1	(<0.1)	96	(25.3)	279	(73.4)	5	(1.3)		
Informal lender	3103	(61.6)	1766	(35.0)	170	(3.4)	1	(<0.1)	106	(27.9)	271	(71.3)	3	(0.8)		
Friends or relatives	4620	(91.7)	305	(6.1)	114	(2.3)	1	(<0.1)	262	(69.0)	111	(29.2)	7	(1.8)		
Group based microfinance	1758	(34.9)	3141	(62.3)	140	(2.8)	1	(<0.1)	288	(75.8)	89	(23.4)	3	(0.8)		
Informal credit/savings group	1201	(23.8)	3713	(73.7)	125	(2.5)	1	(<0.1)	364	(95.8)	15	(4.0)	1	(0.3)		

a Schooling level not available for female respondents in BRB dataset.

### 4.2 Preliminary inspection of agency item sets

Table 3 shows the distributions of baseline responses in the TRAIN and BRB samples with respect to intrinsic agency item sets considered for IRT models. For *intrinsic agency in the right to bodily integrity*, 1.1% or fewer values were missing for any item, and responses were adequately distributed across response options. In TRAIN, the prevalence of justifying IPV ranged from 5.0% to 26.9% across situations (items). In BRB, this prevalence ranged from 21.7% to 56.3% across items. Qualitatively, women justified IPV more often when a wife argues with her husband than if she burns the food.

**Table 3:** Percentages of responses for Intrinsic Agency Items, Pro-WEAI Baseline Survey, Women Participating in TRAIN Bangladesh and BRB Burkina Faso GAAP2 Projects.

	TRAIN, Bangladesh (N = 5040)							BRB, Burkina Faso (N = 380)						
**Bodily Integrity** Is a husb. justified in hitting his wife if…?					Yes	No	Missing					Yes	No	Missing
She goes out without telling him					17.4	82.5	0.1					43.7	55.5	0.8
She neglects the children					17.4	82.1	0.5					43.7	55.8	0.5
She argues with him					26.9	72.9	0.2					56.3	43.2	0.5
She refuses to have sex with him					6.4	93.5	0.2					35.1	63.8	1.1
She burns the food					5.0	94.4	0.6					21.7	77.8	0.5
**Autonomy in Income** How similar are you to s.o. who…?			Com Same	Som Same	Som Diff	Com Diff	Missing			Com Same	Som Same	Som Diff	Com Diff	Missing
Has no alternative to how she can use her income. How she uses her income is determined by necessity			61.6	15.1	9.1	14.2	0.2			14.2	7.2	18.0	60.6	0.0
Uses her income how her spouse or another person or group in her community tell her to			57.5	14.6	12.9	15.0	<0.1			26.3	23.1	17.4	33.2	0.0
Uses her income how her family or community expects because she wants them to approve of her			63.1	16.9	10.6	9.4	<0.1			15.6	10.2	20.1	54.2	0.0
Chooses to use her income how she wants to and thinks is best for herself and her family			70.5	15.7	3.6	10.3	<0.1			61.4	21.5	7.2	9.9	0.0
**Livelihood activities** To what extent do you feel you can take part in decisions about…?^a^		High	Med	Small	Not at all	No Part	Missing		High	Med	Small	Not at all	No Part	Missing
Staple grain farming		25.8	30.7	18.9	11.0	13.6	0.0		37.1	35.3	9.5	16.1	0.3	1.8
High value crop farming		8.9	5.6	3.3	1.5	80.8	0.0		25.5	16.8	4.7	4.7	47.1	1.1
Raising large livestock		23.3	17.4	10.3	4.7	44.3	0.0		16.1	19.7	8.4	19.7	35.8	0.3
Raising small livestock		15.2	8.6	4.2	2.5	69.7	0.0		48.2	23.7	9.0	9.2	10.0	0.0
Raising poultry		60.1	12.4	7.2	3.3	17.0	0.1		32.9	25.3	8.4	14.5	18.7	0.3
Fishpond culture		2.1	1.9	1.5	1.3	93.3	0.0		0.5	0.3	0.0	0.3	99.0	0.0
Non-farm economic activities		5.1	4.1	2.0	1.1	87.7	0.0		50.8	5.0	0.5	1.1	42.6	0.0
Wage and salary employment		3.6	2.3	1.5	1.4	91.2	0.0		17.1	1.1	0.3	0.8	80.8	0.0
Occasional large household purchases		10.7	11.0	8.6	3.3	66.4	0.0		35.3	32.9	10.0	21.6	0.0	0.3
Routine household purchases		40.5	27.4	14.9	4.1	13.1	0.0		38.4	35.0	7.4	19.2	0.0	0.0
**Group Membership** To what extent do you feel you can influence decisions in…group?^a^	High	Med	Small	Not at all	No Part	No Grp	Missing/DK	High	Med	Small	Not at all	No Part	No Grp	Missing/DK
Agriculture/livestock	0	<0.1	0.1	0	9.0	80.6	10.1	32.4	26.8	4.6	0.8	22.0	12.3	1.1
Water users	0.1	0.1	0	0.1	3.3	87.6	8.9	3.8	2.4	0.8	0	25.2	57.6	10.2
Forest users	0	0	0	0	0.5	90.7	8.8	1.9	1.1	0.3	0	24.9	53.4	18.5
Credit or Microfinance	2.6	5.9	9.6	5.8	20.2	50.8	5.0	28.2	28.7	4.8	0.8	25.5	9.9	2.1
Mutual help/insurance	0	<0.1	0.1	<0.1	4.7	84.8	10.4	13.7	16.9	3.0	0	7.2	52.8	6.4
Trade/business association	<0.1	0.1	<0.1	0	11.5	77.1	11.2	5.6	3.5	1.6	0	7.5	62.7	19.0
Civic	0	0.1	<0.1	0	2.2	84.9	12.8	9.7	12.9	3.5	0.5	15.0	44.2	14.2
Religious	0.3	2.0	2.0	0.4	25.0	60.9	9.4	26.0	23.6	7.8	1.3	30.0	8.6	2.7

Notes. Com = completely; Som = somewhat; No Part = did not participate; No Grp = no group in community. Don’t know responses were allowed, but were not reported.

a Women who did not participate or without a group in the community were skipped out of answering question(s) regarding felt ability to participate in decisions.

For *intrinsic agency in livelihoods activities*, very few responses were missing for any item in TRAIN, and less than 1.8% of responses were missing for items in BRB. As expected, the percentage of women who did not participate in agricultural activities varied by activity. In TRAIN and BRB, more than 90% of women reportedly did not participate in fishpond agriculture, and more than 80% did not participate in wage and salary employment. Among women in TRAIN, a majority did not participate in high-value crop farming, raising small livestock, non-farm economic activities, and occasional large household purchases. For non-participating women, questions were not asked about the extent they felt they could participate in decisions about these activities. For women who reported participating in specific livelihoods activities, the distributions of their responses about felt capacity to influence decisions varied across activities (*intrinsic agency in livelihoods activities*). In both samples, a majority of women felt they could participate to a medium or high extent in decisions about staple grain farming, raising poultry, and routine household purchases. In BRB, a majority of women felt they could participate to this extent in decisions about raising small livestock, non-farm economic activities, and occasional large household purchases. A majority of participating women in TRAIN felt they could participate to any (small, medium, high) extent in decisions about raising large livestock. In both samples, among the minorities of women who participated in customarily male-dominated livelihoods activities, a majority felt they could participate to a medium or high extent in decisions about those activities. Thus, higher *intrinsic agency in livelihoods activities* was related to whether or not women participated in the activity at all.

Regarding *autonomy in income*, none of these questions were filtered by skip patterns, and little to no data were missing for other reasons in both samples. In TRAIN, a majority of women reported that they were somewhat or completely like others who used their income according to necessity or how others told them or expected them to (three items); however, a majority of women also reported that they were somewhat or completely like others who chose to use their income how they wanted to (one item). In BRB, a majority of women consistently reported being somewhat or completely like others who used their income as they chose and somewhat or completely different from others who used their income according to necessity or how others told or expected them to.

Regarding *intrinsic agency to influence group decisions*, most women in TRAIN reported that either the group was not present or they were not an active member, so follow-up questions about felt ability to influence group decisions were not asked. In BRB, high percentages of women had either missing data for presence of the group (mostly reflecting ‘don’t know’ responses),^13^ or reported no group or non-participation in the group. For women in BRB who reported being an active group member, the majority felt they could influence decisions to a medium or high extent.

Regarding the item set designed to capture *instrumental agency in livelihoods activities* (Table 4), the extent of missingness and (by design) non-participation were similar to the item set for *intrinsic agency in livelihoods activities*. Among women who reported participating in specific livelihoods activities, the majority reported engaging in some or most/all of the decisions for that activity. Participating women also reported engaging in some or most/all decisions regarding the outputs and income generated from specific livelihoods activities. Thus, within both samples, substantial similarities were observed in the distributions of responses for item sets designed to capture *intrinsic and instrumental agency in livelihoods activities*. Among women who participated in each activity, there was a tendency to report a medium/high extent of intrinsic agency and some/most-all input in decisions, or instrumental agency.

**Table 4:** Percentages of Responses for Instrumental Agency Items, Pro-WEAI Baseline Surveys, Women Participating in TRAIN Bangladesh and BRB Burkina Faso GAAP2 Projects.

	TRAIN, Bangladesh (N = 5040)					BRB, Burkina Faso (N = 380)				
**Livelihood activities**: How much input did you have in making decisions around…?^a^	Most/all	Some	Little/none	No Part	Missing	Most/all	Some	Little/none	No Part	Missing
Staple grain farming	31.2	41.1	11.7	16.1		42.9	37.4	16.8	0.8	2.1
High value crop farming	10.1	6.8	2.0	81.2		27.1	19.7	5.0	47.1	1.1
Raising large livestock	27.1	21.3	5.6	46.0		16.3	24.5	23.2	35.8	0.3
Raising small livestock	17.1	9.9	2.6	70.5		50.3	28.7	11.1	10.0	0.0
Raising poultry	62.4	14.8	4.3	18.4	0.1	34.2	29.7	17.1	18.7	0.3
Fishpond culture	2.3	2.9	1.1	93.8		0.5	0.3	0.3	99.0	0.0
Non-farm economic activities	6.2	4.5	1.4	87.9		51.3	4.7	1.3	42.6	0.0
Wage and salary employment	4.6	2.7	1.3	91.4		17.1	1.3	0.8	80.8	0.0
Occasional large household purchases	12.9	16.1	3.7	67.3		37.1	36.6	25.5	0.5	0.3
Routine household purchases	46.4	34.2	5.4	14.0		40.0	35.8	24.2	0.0	0.0
**Outputs**: How much input did you have in how much to keep rather than sell?^a^	Most/all	Some	Little/none	No Part	Missing	Most/all	Some	Little/none	No Part	Missing
Staple grain farming	34.1	38.6	11.2	16.1	<0.1	42.9	37.4	16.8	0.8	2.1
High value crop farming	10.0	6.3	2.1	81.6		27.1	19.7	5.0	47.1	1.1
Raising large livestock	25.7	21.3	6.2	46.9		16.3	24.5	23.2	35.8	0.3
Raising small livestock	15.3	10.2	2.9	71.8		50.3	28.7	11.1	10.0	0.0
Raising poultry	59.9	15.9	4.4	19.7		34.2	29.7	17.1	18.7	0.3
Fishpond culture	2.4	2.5	1.3	93.8		0.5	0.3	0.3	99.0	
**Income:** How much input did you have in deciding how to use income from…?^a^	Most/all	Some	Little/none	No part	Missing	Most/all	Some	Little/none	No part	Missing
Staple grain farming	31.2	40.5	11.8	16.6	<0.1	42.1	39.2	18.4	0.3	0.0
High value crop farming	9.7	6.3	2.2	81.8		26.8	21.1	5.0	47.1	0.0
Raising large livestock	24.6	22.0	6.3	47.1		16.6	25.3	22.4	35.8	0.0
Raising small livestock	15.1	10.5	2.8	71.6		47.9	30.5	11.6	10.0	0.0
Raising poultry	59.6	15.7	4.5	20.1		34.0	29.5	17.9	18.7	0.0
Fishpond culture	2.4	2.4	1.3	94.0		0.3	0.5	0.3	99.0	0.0
Non-farm economic activities	5.9	4.9	1.4	87.8		50.0	5.8	1.3	42.4	0.5
Wage and salary employment	4.4	3.1	1.1	91.3		16.8	1.6	0.8	80.8	0.0
**Borrowing**: Who made the decision to borrow from…most of the time?^a^^,^^b^		Part inv	Part not inv	HH not inv	Missing		Part inv	Part not inv	HH not inv	Missing
NGO		61.6	29.5	8.9	<0.1			19.3	80.7	
Formal lender		5.9	3.9	90.2	<0.1			5.4	94.6	
Informal lender		6.0	3.1	90.9	<0.1			8.3	91.7	
Friends or relatives		20.2	10.7	69.0	<0.1			33.8	66.2	
Group-based microfinance		4.4	2.8	92.8	<0.1			44.5	55.5	
Informal credit group		0.7	0.5	98.8	<0.1		0.3	63.5	36.2	
**Borrowing**: Who made the decision about what to do with money from…most of the time?^a^^,^^b^		Part inv	Part not inv	HH not inv	Missing		Part inv	Part not inv	HH not inv	Missing
NGO		52.6	38.5	8.9	<0.1			19.3	80.7	
Formal lender		5.0	4.8	90.2	<0.1			5.4	94.6	
Informal lender		5.2	3.9	90.9	<0.1			8.3	91.7	
Friends or relatives		17.3	13.7	69.0	<0.1			33.8	66.2	
Group-based microfinance		3.8	3.4	92.8	<0.1			44.5	55.5	
Informal credit group		0.6	0.5	98.8	<0.1		0.3	63.5	36.2	
**Borrowing**: Who was responsible for repaying the money borrowed from…?^a^^,^^b^		Part inv	Part not inv	HH not inv	Missing		Part inv	Part not inv	HH not inv	Missing
NGO		33.8	57.3	8.9	<0.1			17.2	80.7	2.1
Formal lender		4.6	5.2	90.2	<0.1			3.5	94.6	1.9
Informal lender		3.8	5.3	90.9	<0.1			6.7	91.7	1.6
Friends or relatives		12.8	18.2	69.0	<0.1			31.9	66.5	1.6
Group-based microfinance		3.4	3.8	92.8	<0.1			44.2	55.5	0.3
Informal credit group		0.5	0.7	98.8	<0.1		0.3	62.7	36.2	0.8

Notes. No Part = Did not participate; Part inv = participant involved; Part not inv = participant not involved; HH no inv = household not involved, either because the household was unable to borrow from the specific source and whether the household did not borrow from this source in the prior 12 months.

a Women who did not participate or whose household was not involved were skipped from the question(s) that asked about involvement or input into decisions.

b The question refers to the year prior to survey and is asked of respondents who reported that their household had taken a loan or borrowed cash/in kind from that entity during the last year.

Finally, regarding women’s *instrumental agency in borrowing from financial services*, women generally reported that their households were not involved in borrowing money from specific sources (especially in TRAIN). When respondents reported that someone from their household borrowed money from an institution or group within the last year, a minority of women in TRAIN reported being involved in decisions about borrowing, and most women in BRB reported not being involved in these decisions.

### 4.3 Evaluating the assumption of unidimensionality

As a next step, we evaluated the assumption of unidimensionality by fitting a one-factor CFA to each intrinsic agency item set with low missingness and binary or ordered response options (*intrinsic agency in the right to bodily integrity*, *autonomy in use of income*). Full results are available on request. For the *intrinsic agency in the right to bodily integrity* items, unidimensional CFA models fit the data well in both samples and were adequate for conducting unidimensional IRT analysis (TRAIN CFI = 1.000, TLI = 1.000, RMSEA = 0.009 90% CI [0.001, 0.023]; BRB CFI = 0.997, TLI = 0.995, RMSEA = 0.041, 90% CI [0.000, 0.090]). Results for the CFAs of *autonomy in use of income* showed adequate model fit in TRAIN (CFI = 1.000, TLI = 1.000, RMSEA = 0.012 90% CI [0.001, 0.033]) but poor model fit in BRB (CFI = 0.945, TLI = 0.835, RMSEA = 0.095 90% CI [0.038, 0.164]). Thus, further results for *autonomy in use of income* are not presented.

### 4.4 Evaluating the assumption of local independence

As a next step, for each estimated IRT model, we evaluated the assumption of local independence using the LD *Χ*^2^ statistic for item pairs in sets for which the assumption of unidimentionality was met in CFA or response options were nominal (and CFA was not estimated). Table 5 summarizes these statistics according to threshold values (see Methods). For the IPV-attitudes item set, all LD Χ^2^ statistics in both samples provided evidence of local independence (<|5|). For all other item sets, between 80% and 100% of LD *Χ*^2^ statistics in TRAIN and between 39% and 100% of LD X^2^ statistics in BRB provided evidence of questionable or probable local dependence (≥|5|). Some LD X^2^ could not be computed by IRTPRO, which may be caused by the smaller sample size of BRB. Thus, most item sets measured in these two samples that are the basis of pro-WEAI indicators displayed substantial pairwise dependence beyond the hypothesized latent construct, a violation that can adversely affect model estimates, model-fit statistics, and derived scores. For this reason, we present hereafter IRT model estimates and fit statistics only for the IPV-attitudes items.^14^

**Table 5:** Distribution of Standardized LD X2 Statistics by Recommended Threshold, Tests for Local Dependence for Pairwise Agency Items, pro-WEAI Baseline Surveys, Women Participating in TRAIN Bangladesh and BRB Burkina Faso GAAPs Projects.

	TRAIN, Bangladesh (N = 5040)			BRB, Burkina Faso (N = 373)		
Number of item pairs for which:	LD X^2^ < |5| (local dependence unlikely)	|5| ≤ LD X^2^ ≤ |10| (local dependence possible)	LD X^2^ > |10| (local dependence probable)	LD X^2^ < |5| (local dependence unlikely)	|5| ≤ LD X^2^ ≤ |10| (local dependence possible)	LD X^2^ > |10| (local dependence probable)
**Intrinsic Agency**						
Bodily Integrity (5 IPV attitudes items; 10 LD statistics)	10	0	0	10	0	0
Autonomy in Income (4 RAI items; 6 LD statistics)	0	0	6	0	2	4
Livelihoods activities (10 items; 45 LD statistics)^a^	1	6	38	20	5	11

**Instrumental Agency**						
Livelihoods activities (10 items; 45 LD statistics)^a^	6	13	26	22	4	10
Sale/use of outputs (6 items; 15 LD statistics)^b^	2	5	8	2	2	7
Use of income (8 items; 28 LD statistics)^b^	5	9	14	11	6	7
Borrowing from financial services (6 items; 15 LD statistics)^c^	3	3	9	–	–	–

a Nine LD X2 statistics not estimated for BRB by IRTPRO.

b Four LD X2 statistics not estimated for BRB by IRTPRO.

c Not estimated for BRB as there was little variability, and only the model with a subset of the Borrowing converged.

### 4.5 Assessing model fit

Table 6 presents model estimates and item-level fit statistics (S-*Χ*^2^) for IRT analyses of all five IPV-attitudesitems (Panel 1) and a subset (Panel 2). In Panel 1, S-*X*^2^ statistics indicate a satisfactory fit for all five IPV-attitudes items in BRB and for three of the five items in TRAIN. The items ‘she refuses to have sex with him’ and ‘she burns the food’ showed poor model-data fit at the item level in TRAIN. To address this issue, we removed the item ‘she burns the food,’ which had the poorest fit (the highest S-X^2^ value) and re-estimated the model (Panel 2). Based on S-X^2^ test statistics, model-data fit for the four remaining items was good in both samples.

**Table 6:** Assessment of Model Fit, 2PL Item-Response Model for Intrinsic Agency in Bodily Integrity (IPV-Attitudes Item Set), Women Participating in Baseline pro-WEAI Survey in TRAIN Bangladesh and BRB Burkina Faso GAAP2 Projects.

	TRAIN, Bangladesh (N = 5040)							BRB, Burkina Faso (N = 373)						
**Panel 1 (5 items)**														
Is a husband justified in hitting his wife if…?	a	SE_a_	b	SE_b_	*X*^2^	df	Prob	a	SE_a_	b	SE_b_	*X*^2^	df	Prob
She goes out without telling him	3.34	0.18	−1.07	0.03	3.40	3	0.3300	3.34	0.18	−1.07	0.03	3.26	3	0.3550
She neglects the children	3.48	0.19	−1.06	0.03	2.44	3	0.4874	3.48	0.19	−1.06	0.03	4.87	3	0.1827
She argues with him	4.51	0.33	−0.66	0.02	0.18	2	0.9148	4.51	0.33	−0.66	0.02	2.17	3	0.5387
She refuses to have sex with him	2.47	0.15	−1.88	0.05	11.14	3	0.0110	2.47	0.15	−1.88	0.05	2.25	3	0.5233
She burns the food	2.35	0.15	−2.07	0.06	27.90	3	0.0001	2.35	0.15	−2.07	0.06	3.67	3	0.3007

**Panel 2 (4 items: she burns the food removed)**														
Is a husband justified in hitting his wife if…?	a	SE_a_	b	SE_b_	*X*^2^	df	Prob	a	SE_a_	b	SE_b_	*X*^2^	df	Prob
She goes out without telling him	2.99	0.36	−0.40	0.72	0.33	2	0.8462	2.43	0.39	−0.18	0.08	0.71	2	0.7006
She neglects the children	3.07	0.35	−0.40	0.73	2.02	2	0.3654	3.28	0.62	−0.18	0.07	1.00	2	0.6057
She argues with him	4.00	0.37	0.05	0.76	0.18	2	0.9136	2.54	0.42	0.20	0.08	1.68	2	0.4321
She refuses to have sex with him	2.22	0.28	−1.32	0.67	0.62	2	0.7331	2.29	0.38	−0.46	0.08	0.61	2	0.7365

For all other item sets (not shown), all or nearly all items exhibited significant S-X^2^ values, providing strong evidence that model-predicted and observed response frequencies differed. We experimented with removing items having the highest S-X^2^ values, but model-data fit improved little, and the assumption of local independence remained untenable (results available on request). Consequently, we continue to present results only for the four IPV-attitudes items for which the assumptions of unidimensionality, local independence, and model-data fit at the item level were met. We then discuss, with illustrative graphs, some challenges of interpretation regarding results for the other item sets for which model assumptions were not met. We focus on graphs for selected items and item sets for *intrinsic* and *instrumental agency in livelihoods activities* and discuss possible reasons for the challenges of interpretation they expose.

### 4.6 Comparing competing models

In our next step for the analysis of the four IPV-attitudes items, we compared the posited 2PL IRT model having a separate discrimination parameter for each item to a 1PL IRT model, where the discrimination parameter was fixed at one across all four items. The AIC and BIC were larger in the alternative 1PL IRT model (AIC = 15377.10, BIC = 15456.26) than in the original 2PL IRT model (AIC = 15343.41, BIC = 15462.14). This finding suggests that the more parsimonious common-slope model was insufficient to capture the extent of cross-item heterogeneity in discrimination parameters.

### 4.7 Evaluating and interpreting results

**Fig. 2:**
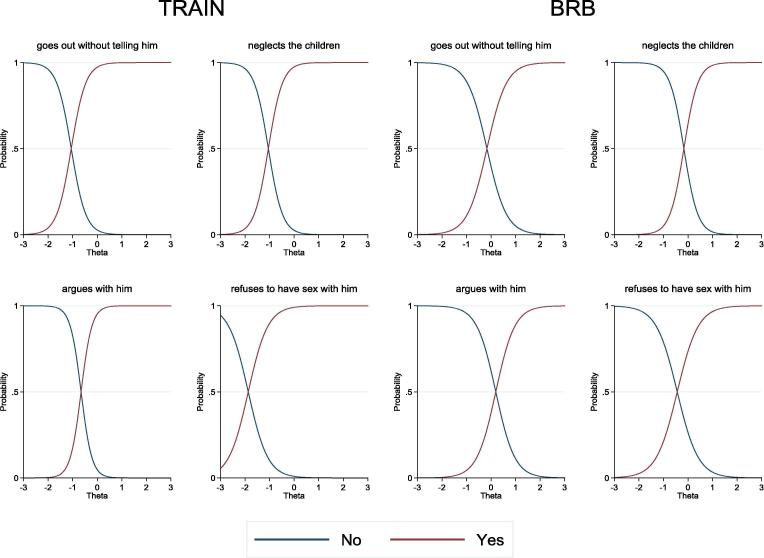
A matrix plot of item characteristic curves for *Intrinsic Agency in Bodily Integrity* (four IPV attitudes items from Table 6, Panel 2), TRAIN and BRB projects.

To assess and interpret results of the final, four IPV-attitudes items 2PL IRT model, we relied on item characteristic curves (ICCs), item information curves (IICs) and total information curves (TICs). Fig. 2 shows a matrix plot of the ICCs for the four IPV-attitudes items from model estimates shown in Table 6, Panel 2. The value of theta where the ICCs intersect with one another in each graph gives the estimate of the difficulty parameter for each item. Consistent with descriptive findings in Section 4.2, the ICCs show that the item ‘she argues with him’ is the most difficult one to answer ‘not justified’ in both samples. The slope of the item ‘she argues with him’ also is the steepest and most discriminating item of the four for TRAIN. For BRB, the slope of item ‘she neglects the children’ is the most discriminating.

**Fig. 3:**
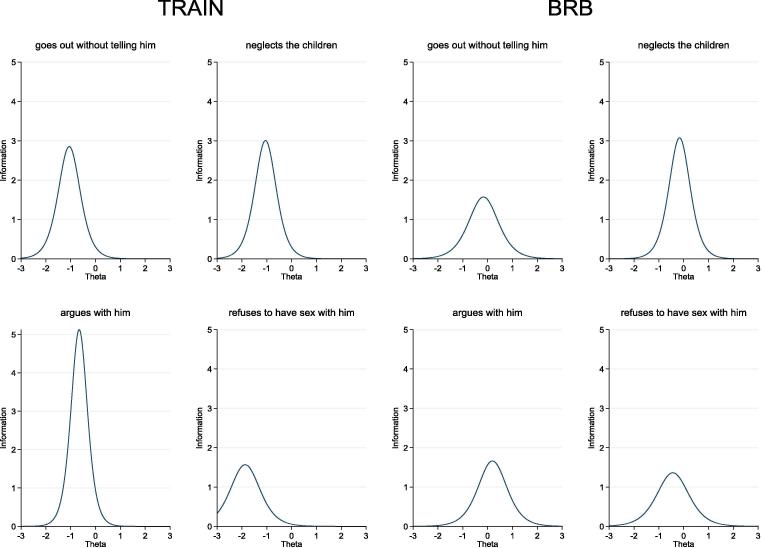
Item information functions for *Intrinsic Agency in Bodily Integrity* (four IPV-attitudes items from Table 6, Panel 2), TRAIN and BRB projects.

**Fig. 4:**
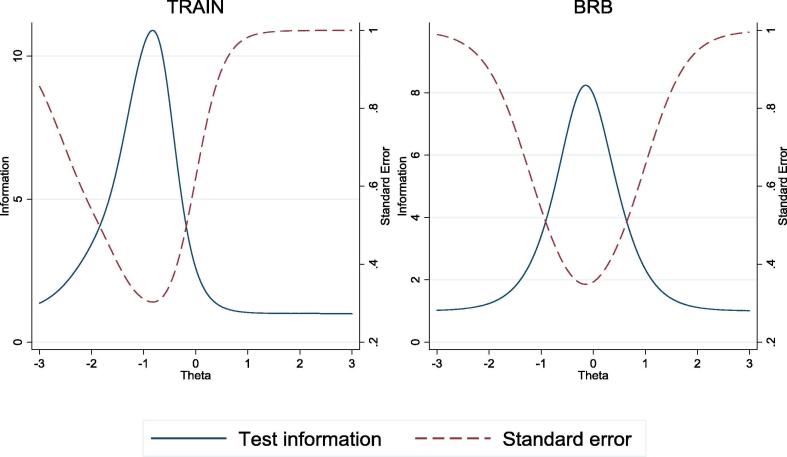
Total information curves for *Intrinsic Agency in Bodily Integrity* (four IPV-attitudes items from Table 6, Panel 2), TRAIN and BRB projects.

Fig. 3 displays a matrix plot of the item information curves. The graphs show that ‘she neglects the children’ for the BRB sample and ‘she argues with him’ for the TRAIN sample provide maximum precision around the mean level of the latent trait, where Θ = 0. Fig. 4 presents the total information curves for the same four IPV-attitudes items for both samples. Both curves suggest that the item set provides more precision around the mean level of the latent-agency trait, where Θ = 0, and less precision at higher and lower levels of the latent trait. The TICs for both samples also are similar to one another, which suggests that these four IPV-attitudes items provide similar precision across the two samples.

### 4.8 Assessing measurement equivalence

Because model assumptions (of unidimensionality, local independence, and model fit) were met in both samples only for the four IPV-attitudes items, we limited our assessment of cross-sample measurement equivalence to this item set. We investigated whether any of the four items displayed differential item functioning (DIF), or whether estimates of the discriminations (a parameters) and the difficulties (b parameters) differed across the two samples, holding constant the level of the latent-agency trait (see Cheong et al. (nd) for more detail). We detected two items with DIF across TRAIN and BRB, a husband is justified to beat his wife if ‘she argues with him’ and ‘she refuses to have sex with him.’ However, the impact of DIF on the mean difference in the agency scores was small, 0.06, such that the four items were considered for practical purposes to have measurement equivalence across the two samples.

### 4.9 Considerations for interpretation of other item-sets in the pro-WEAI survey

As discussed, IRT estimates for other item sets in the pro-WEAI survey showed that the assumption of local independence was untenable and model-data fit was poor. As a result, the model parameter estimates and their standard errors were likely distorted. Here, we present some graphical results of the functional forms of the nominal response models for the item sets designed to capture *instrumental agency in livelihood activities* (10 items), *instrumental agency in the sale or use of outputs and use of income* (14 items), and *intrinsic agency in livelihood activities* (10 items). The graphical displays are illustrative only and offer tentative reasons on how the items behaved.

**Fig. 5:**
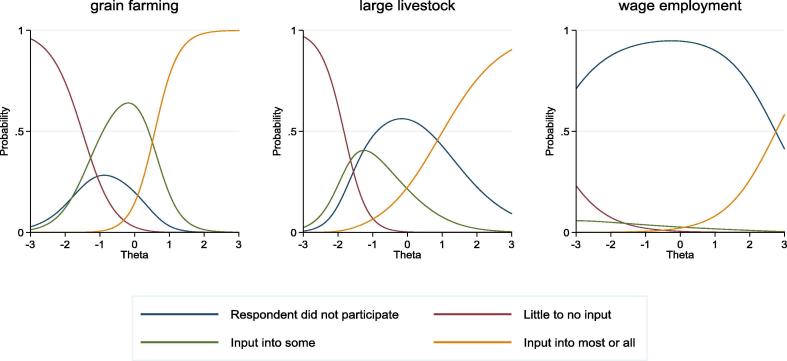
Category characteristic curves for nominal response models for *Instrumental Agency in Livelihoods Activities* for activities with a low (Grain Farming), moderate (Large Livestock), and high (Wage Employment) level of non-participation, TRAIN project.

First, we examined category characteristic curves (CCCs) for items tapping *instrumental agency in livelihood activities*.^15^ In Fig. 5, three distinct patterns in the CCCs can be observed that correspond to different levels of women’s participation in livelihood activities. First, respondents tended to report non-participation over a small range of the latent trait of *instrumental agency in livelihood activities* for grain farming (shown in Fig. 5), poultry, and routine household purchases. Second, respondents tended to report non-participation over a moderate range of the instrumental agency latent trait for large livestock raising. Third, respondents tended to report non-participation over a full range of the latent trait for wage employment (shown in Fig. 5), horticulture, fishpond, small livestock and large household purchases. This patterning in the response options may be illustrative of the non-ordered nature of the response options and the heterogeneous latent agency of women who report non-participation in some livelihoods activities.

**Fig. 6:**
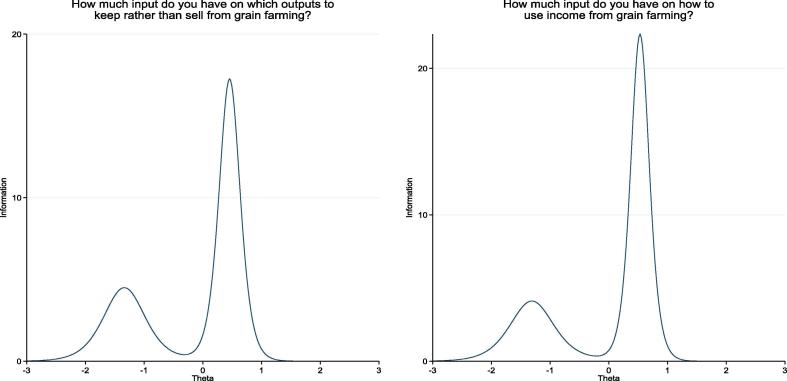
Item information curves for nominal response models for *Instrumental Agency in the Use or Sale of Outputs* and *Instrumental Agency in the Use of Income* for grain farming, TRAIN project.

Next, we examined the item information curves of the 14 items for *instrumental agency in the sale or use of outputs*/*income* generated. Fig. 6 illustrates the IICs for grain farming in TRAIN. As in Fig. 6, for items that asked respondents to report the keeping-not-selling and income use decisions on the same activities, their item information curves were very similar (full set of IICs available on request). This result may suggest that either item set on sale or use of outputs/income may be dropped with little loss of precision. It might also indicate that the participants did not differentiate the two sets of decisions for the same livelihoods activities in their responses.

**Fig. 7:**
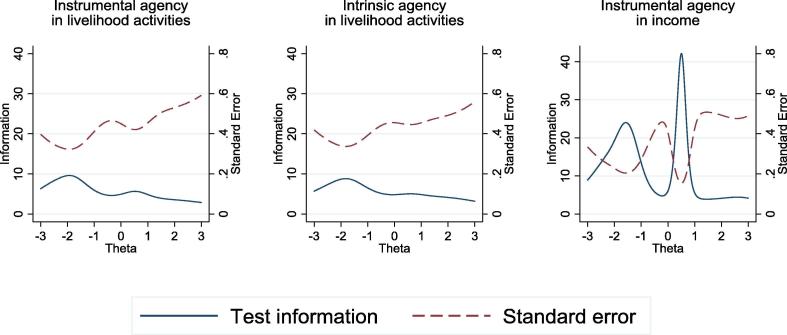
Total information curves for nominal response models for *Instrumental Agency in Livelihoods Activitie*s, *Intrinsic Agency in Livelihoods Activities*, and *Instrumental Agency in the Use of Outputs/Income*, TRAIN project.

Finally, we examined the test information functions for each of the three item sets that captured *intrinsic agency in livelihoods activities*, *instrumental agency in livelihoods activities*, *instrumental agency in the sale or use of outputs and use of income* (Fig. 7). As Fig. 7 shows, the precision of each item set along the latent-agency-trait continuum is similar to that of the other two sets. Thus, item sets that are used to construct the same pro-WEAI indicator (Appendix 2) could be dropped without a loss of precision.

## 5 Discussion

This analysis is the first to use IRT methods to assess the measurement properties of a women’s empowerment scale in development studies. It also is the first to assess the measurement properties of item sets that form the basis of indicators in pro-WEAI, an instrument in the WEAI series designed to assess women’s empowerment in agriculture and more generally. The methodological innovations applied here provide a guide for development researchers to design, test, and refine questionnaires that include item sets aiming to capture women’s intrinsic, instrumental, and collective agency in agricultural and other development programs designed to empower women.

### 5.1 Findings and implications

A relevant descriptive finding from this analysis was that the participation of women in livelihoods activities, financial services, and community-based groups varied across activities, services, and groups as well as across agricultural development projects and contexts. In the case of items designed to capture women’s felt influence in community groups, high levels of non-participation (and the reported absence or lack of knowledge about the presence of community groups) precluded estimation of IRT models. Notably, the BRB program in Burkina Faso was designed to intervene via women’s groups; however, the baseline survey occurred before the project was implemented, so women would not have reported participation in project-related groups. Moreover, the TRAIN project did not involve a group-based intervention, and women in the TRAIN sample were relatively young, and perhaps less likely to participate in community groups. Low reported participation in non-project related groups also may have resulted from interview burden—if interviewers and/or respondents were overwhelmed by the assessment length, not reporting groups or reporting non-participation in groups would have reduced interview time. Alternatively, women may have understood these questions contrary to their intent—and did not report on all groups or all groups in which they were participating or limited their responses only to formal groups in their community, even though informal groups were listed in the questionnaire. Cognitive interviewing would allow us to assess the salience of these considerations for revisions to this module. Also, pro-WEAI might consider questions about forms of collective agency that do not require group membership but instead reflect non-institutional collective action. Candidates for consideration may include survey questions from early versions of the WEAI about women’s engagement in community projects or assistance of other women or families in the community.

Similarly, women’s non-participation was high for some livelihoods activities and financial services. The agency of these women was not measured directly. Some reported non-participation may be related to the use of single key-word questions for certain types of economic activity. For example, six items were included in pro-WEAI to capture women’s participation in agricultural activities; whereas, only one item each was included to capture women’s participation in non-agricultural economic activities and in women’s wage and salary employment. In other studies, single key-word questions have yielded lower rates of economic activity than activity lists (Langsten and Salem, 2008, Yount et al., 2014). Thus, for non-agricultural and wage-based economic activity, high rates of non-participation may, in part, have resulted from using single-key word questions.

Moreover, in other analyses (available on request), women’s non-participation in specific livelihoods activities was differentially associated (positively and negatively) with scores for women’s human, economic, and social resources for empowerment. In other words, the relationships of women’s resources for empowerment to non-participation in specific livelihoods activities varied by the type of resource and livelihoods activity. This finding suggests that non-participants in specific livelihoods activities are heterogeneous with respect to their resources for empowerment. Consistently, boundary characteristic curves from IRT models showed that respondent or household non-participation in specific livelihoods-, income-, or borrowing-related activities was systematically related to the latent-agency trait, and that this relationship differed across items within item sets. Therefore, making assumptions at the indicator-level that systematic non-participants are ‘inadequately’ empowered may warrant further study to rule out misclassification of women who report non-participation in listed activities because they have other resources at their disposal (or because single-key word questions were used for non-agricultural and wage-based activities).

Given these descriptive results, a major finding from this analysis was that one item set—capturing *intrinsic agency in the right to bodily integrity*—met the IRT assumptions of unidimensionality, local independence, model-data fit, and measurement equivalence (also implied in the construction, interpretation, and cross-group comparison of pro-WEAI indicators). This finding confirms a prior validation of these and other IPV-attitudes items (Yount, VanderEnde, Zureick-Brown, Anh, et al., 2014). One caveat of the pro-WEAI item set is that it provides limited precision at the lower and higher ends of the latent intrinsic agency trait, and thus, may have limited capacity to assess change over time. To ensure precise measurement at the extremes of this latent trait, four other validated IPV-attitudes items might be added to the pro-WEAI item set (Yount, VanderEnde, Zureick-Brown, Anh, et al., 2014). Alternatively, response options for current IPV-attitudes items might be expanded to be ordinal, allowing each item to have higher precision across a wider range of the latent-agency trait.

A second major finding was that the remaining item sets did not meet the assumptions of unidimensionality or local independence (LI). For autonomy in income, weak evidence of unidimensionality may have resulted from having items designed to capture different theoretical constructs included in the same set. Consistent with Deci and Ryan, 1985, Deci and Ryan, 1995, Deci and Ryan, 2000), the items, ‘uses her income how her family or community’ ‘tell her she must’ and ‘expect because she wants them to approve of her’ likely capture external motivations in her use of income; whereas, ‘chooses to use her income how she wants to and thinks is best for herself and her family’ likely captures internal motivation, or autonomy. If so, then substituting items that capture external motivation in use of income with items that capture internal motivation in use of income may better reflect the intended unidimensional construct.

For the other item sets, strong evidence of local dependence may be problematic for interpretation of derived indicator values. Again, LI means that the latent trait variable being measured is the only influence on a woman’s response to an agency item; thus, for a given woman with a known agency score, her response to one item should be independent of her response to any other item. Empirically, evidence of local dependence means that model estimates, model fit statistics, and derived scores and associated standard errors may be distorted and may differ from the theoretical construct being measured (Toland, 2014). LD can occur when items or questions within a set sound similar to respondents, who then repeatedly provide the same answer. The many matrices in the pro-WEAI—in which multiple questions are asked of lists of items—corroborates this interpretation. In practice, women who do not participate in an activity are not asked questions about that activity in pro-WEAI, so are not asked the full matrix. However, respondents may not distinguish similar questions asked of the same activity, resulting in similar answers to questions designed to tap different theoretical constructs. Cognitive interviewing of these matrices may identify clearer wording to minimize this possible source of LD.

A third major finding was the high overlap in total information curves for item sets designed to capture distinct agency constructs. The TICs for *instrumental agency in livelihoods activities* and *instrumental agency in the use of income* were illustrative. These findings suggest that one or the other item sets could be dropped from pro-WEAI without a substantial loss of precision in measuring the latent-agency-trait continuum. Alternatively, pro-WEAI modules could be revised to enhance the distinctiveness of item-sets for respondents. Modules could begin with a more detailed introduction clarifying the purpose of new question sets. Item sets could begin with a warm-up to ensure correct interpretation. Questions in multiple-question matrices could be revised to ensure distinctiveness for respondents. However, if respondents do not, in practice, make fine distinctions between types of agency, then avoiding question sets that seek nuance between types of agency may be advised.

### 5.2 Limitations and strengths of the analysis and pro-WEAI

Some caveats of the analysis are notable. First, TRAIN and BRB implemented slightly different versions of the pro-WEAI questionnaire, and not all modules were asked in both countries. Second, interview duration varied across projects, on average, requiring one hour in TRAIN and one hour forty minutes in BRB (which included the health and nutrition add on). Despite variations in average interview duration, results of the analysis were broadly consistent. Third, although we aimed to validate the collective agency item set, the IRT estimation was not possible because the item set focused on felt influence in group decisions among active members. As discussed, most women reported that groups were not known or not present in their community (especially in TRAIN) or that they were not members (especially in BRB). As such, information on felt influence was limited to the few women who reported being active members. Fourth, we were unable to estimate IRT models for instrumental agency in land use, because too few items measured this construct. Fifth, we were unable to assess the measurement equivalence of most item sets across contexts because model assumptions of unidimensionality, local independence, and model-data fit were not met *within* contexts. Finally, we were unable to use EFA/CFA approaches to assess the multidimensionality of item sets for intrinsic and instrumental agency because of several partially ordered item sets. To address this limitation, we removed items with high non-participation (>60%), because these items require nominal IRT models. We, then, conducted a series of multidimensional EFAs of retained items intended to capture distinct but correlated dimensions of intrinsic agency, and separately, distinct but correlated dimensions of instrumental agency. We conducted this analysis with TRAIN data only, because too many items were dropped for BRB. One-to-five factor EFA models were estimated in a random split sample to allow for CFA, if EFA suggested a CFA was warranted. For instrumental agency, items loaded by *livelihood activity*, not by *instrumental agency construct*, suggesting again that respondents interpreted similarly questions that were intended to capture different aspects of instrumental agency for each activity. For intrinsic agency, items loaded by expected construct (general self-efficacy items loaded on the same factor; autonomy in income, etc). Fit indices also were good. However, except for previously validated scales (self-efficacy and autonomy in income, specifically), the pro-WEAI dimensions of intrinsic agency generally were not highly correlated with the other dimensions (results available on request). Thus, in this sub-sample for TRAIN, the subset of items with higher percentages of participating women did not appear to be tapping correlated, multidimensional constructs of instrumental agency, and in turn, intrinsic agency. Thus, work remains to operationalize the constructs of women’s intrinsic and instrumental agency in agriculture. After refining the pro-WEAI instrument to address these issues, we suggest that this validation be reapplied to the revised pro-WEAI long-form to confirm that item sets align with their intended theoretical constructs (or indicators) within contexts. Then, the measurement equivalence of pro-WEAI item sets across contexts, genders, and time can be assessed, and a valid short-form version can be identified.

A sixth caveat of the analysis was its application to 2 of the 13 GAAP2 projects, so findings are generalizable only to the TRAIN and BRB samples. Validation of the revised pro-WEAI ideally will occur across more projects, contexts, and genders. Finally, the analysis focuses on the measurement properties of survey items in pro-WEAI and does not fully account for the aggregation methodology used for constructing pro-WEAI indicators (e.g., setting adequacy thresholds and censoring headcounts); thus, we cannot comment definitively about the implications of the findings for the overall calculation of pro-WEAI.

These caveats notwithstanding, the many strengths of this analysis are notable. IRT methods are powerful techniques to validate instruments, like pro-WEAI, within and across settings. Results can help researchers to target questionnaire refinements, such as dropping redundant questions or revising poorly functioning questions to improve clarity. IRT methods also are useful to identify precise (and theoretically sound) item subsets for use in short-form versions of validated long forms. Nominal item-response models can test assumptions about the ordering of polytomous response options. Ordered polytomous response options provide additional information on a respondent’s quantity of the latent trait; however, binary response options could provide similar information with less complexity. These uses can improve instrument quality, reduce respondent burden, and improve the data collected. Finally, this IRT analysis is the first to outline a clear process for researchers and evaluators to assess the measurement properties of any major instrument to measure women’s empowerment. We urge all researchers to use these methods in the first phase of instrument development to ensure that tools recommended for monitoring and evaluation of development programs are empirically sound and consistent with theory. This analytic approach sets the standard for developing and validating measures of women’s empowerment going forward. Notably, the software required to assess the dimensionality of nominal IRTs and to estimate multidimensional IRTs is evolving. The utility of different IRT software packages is presented elsewhere (Cheong et al., nd).

The strengths of pro-WEAI also warrant emphasis. Pro-WEAI is the first instrument designed to measure comprehensively women’s empowerment in agricultural development projects. Its design was based on well-defined theoretical constructs and local knowledge from a diverse set of projects across contexts. The design of pro-WEAI also incorporated learning from efforts to develop the WEAI (Alkire et al., 2013) and other versions (Malapit et al., 2017). Important modifications in pro-WEAI include a more explicit theoretical emphasis on intrinsic, instrumental, and collective agency as well as the creation of a broader set of indicators that allow for a more refined decomposition of changes in women’s agency over a project’s timeline. Tying these strengths with our proposed refinements will improve our capacity to assess the impacts of agricultural development programs on women’s empowerment.

### 5.3 Recommendations for projects

Major takeaways from this analysis are twofold. First, program evaluation would benefit from strategic refinements and shortening of the long-form pro-WEAI and a revalidation following the steps outlined here. Second, program monitoring would benefit from a short-form version of the revised pro-WEAI long-form. Creating a short-form pro-WEAI for monitoring was outside our scope, given our findings that questionnaire refinements are recommended. A short-form pro-WEAI for program monitoring would include simpler question-item sets totaling a 10-minute interview to maximize respondent attentiveness and focus. With a validated long-form and systematically derived short-form, researchers and program managers would be fully equipped to monitor progress and to assess the impacts of agricultural development projects designed to empower women.

## Declaration of Competing Interest

The authors declare that they have no known competing financial interests or personal relationships that could have appeared to influence the work reported in this paper.

## Footnotes

1 A short form is a representative (random) sample of items from a valid long form (Hagtvet & Sipos, 2016) that should be equivalent to or exchangeable with other representative item subsets from the same valid long form (Shavelson & Webb, 1981).

2 Some measures of achievements, such as anthropometric status, may be observed.

3 A supplemental pro-WEAI module asks the primary female decision-maker in each household about decision-making with respect to nutrition and health (Hannan, Heckert, James-Hawkins, & Yount, 2019).

4 Although a prior study showed that this construct was weakly related to other agency constructs, that study used different items to measure attitudes about IPV, so the findings are not comparable (Agarwala & Lynch, 2006).

5 The item, ‘uses her income as determined by necessity,’ is used only for validation and is not used in the construction of the pro-WEAI indicator.

6 This item is not used in the construction of pro-WEAI, and is distinct from the two group-related pro-WEAI indicators, which measure separately women’s active memberships in a group and the degree of influence that a group to which they belong has in the community.

7 Non-participants were skipped out of these follow-up questions on extent of input in decisions.

8 Non-participants were skipped out of these follow-up questions on involvement in decisions.

9 Researchers often desire long questionnaires to measure several constructs. Long questionnaires may produce ‘transient measurement errors’ (Schmidt, Le, & Ilies, 2003) if participants respond carelessly when overwhelmed or bored by the assessment. Such concerns have spurred researchers to create short forms from valid long forms (Stanton, Sinar, Balzer, & Smith, 2002). Researchers should assess whether the short form has comparable within-sample measurement properties to the long form and functions similarly across independent samples (Smith, McCarthy, & Anderson, 2000). Errors creating short forms may arise because guidance for developing them is recent (Marsh, Ellis, Parada, Richards, & Heubeck, 2005; Smith et al., 2000; Stanton et al., 2002) and non-existent in the women’s empowerment literature. Using short forms with poor or poorly understood measurement properties may impede progress toward identifying comparable measures of women’s empowerment to monitor SDG5.

10 Frequently (or easily) endorsed items tend to have lower location parameters on the latent-agency trait; whereas, rarely endorsed (or difficult to endorse) items tend to have higher location parameters on the latent-agency trait.

11 For example, we compared the relative fit of a 2PL model, which estimates the difficulty and discrimination parameters, with a 1PL model, which estimates only the difficulty parameters.

12 The BRB baseline survey did not collect information on women’s schooling.

13 Don’t know responses were not considered missing in pro-WEAI. Reporting that there was no group or that the presence of the group was not known were considered to mean the same thing (Malapit et al., 2019).

14 To illustrate sensitivity analyses, we removed the item “How much input did you have in how much to keep rather than sell? Staple grain farming, whose item pair standardized LD X^2^’s with the other items were considered large (80%) or moderate (20%) for the TRAIN data. We followed Toland’s (2014) recommendations and examined the content of this item and its cross-tabulations with other items on the scale. There were no empty cells in any of the cross-tabulations. A comparison of the parameter estimates with and without the staple grain farming item showed that for the item on raising large livestock, five of the six discrimination and slope parameters changed became at least 1 logit smaller. We performed another analysis for the BRB sample by removing the same item, staple grain farming, whose item pair standardized LD X’s with the other items were considered large (100%). There were no empty cells in its cross-tabulations with the other items. For the item on raising poultry, there were drastic changes of about 8 logits in two of the discrimination and slope parameters after the staple grain farming item was removed, which suggested LD was an issue (results available upon request).

15 We used the irt suite of Stata here (Statacorp, 2017), as it has more graphing flexibility. It does not provide test statistics to check major IRT assumptions. Stata and IRTPRO produce similar graphical summaries.

## References

[b0005] Agarwala R., Lynch S.M. (2006). Refining the measurement of women's autonomy: An international application of a multi-dimensional construct. Social Forces.

[b0010] Alkire S. (2005). Subjective quantitative studies of human agency. Social Indicators Research.

[b0015] Alkire S. (2018). Multidimensional poverty measures as relevant policy tools. https://ophi.org.uk/multidimensional-poverty-measures-as-relevant-policy-tools/.

[b0020] Alkire S., Foster J. (2011). Counting and multidimensional poverty measurement. Journal of Public Economics.

[b0025] Alkire S., Meinzen-Dick R., Peterman A., Quisumbing A., Seymour G., Vaz A. (2013). The women’s empowerment in agriculture index. World Development.

[b0030] Alsop R., Bertelsen M., Holland J. (2006). Empowerment in practice: From analysis to implementation.

[b0035] Bandura A. (2000). Exercise of human agency through collective efficacy. Current Directions in Psychological Science.

[b0040] Bardhan K., Klasen S. (1999). UNDP's gender-related indices: A critical review. World Development.

[b0045] Batliwala S., Sen G., Germaine A., Chen L.C. (1994). The meaning of women’s empowerment: New concepts from action. Population policies reconsidered: Health, empowerment and rights.

[b0050] Beghini, V., Cattaneo, U., & Pozzan, E. (2019). A quantum leap for gender equality: For a better future of work for all. Retrieved from Geneva.

[b0055] Bock R.D. (1972). Estimating item parameters and latent ability when responses are scored in two or more nominal categories. Psychometrika.

[b0060] Bologh R.W. (2009). Love or greatness (Routledge Revivals): Max Weber and masculine thinking.

[b0065] Brody C., de Hoop T., Vojtkova M., Warnock R., Dunbar M., Murthy P., Dworkin S.L. (2017). Can self-help group programs improve women’s empowerment? A systematic review. Journal of Development Effectiveness.

[b0070] Cai L., Du Toit S., Thissen D. (2011). IRTPRO: Flexible, multidimensional, multiple categorical IRT modeling [Computer software].

[b0075] Cai L., du Toit S.H.C., Thissen D. (2011). IRTPRO: User guide.

[b0080] Charmes J., Wieringa S. (2003). Measuring women's empowerment: An assessment of the gender-related development index and the gender empowerment measure. Journal of Human Development.

[b0085] Chen G., Gully S.M., Eden D. (2001). Validation of a new general self-efficacy scale. Organizational Research Methods.

[b0090] Chen S.-F., Wang S., Chen C.-Y. (2012). A simulation study using EFA and CFA programs based the impact of missing data on test dimensionality. Expert Systems with Applications.

[b0095] Chen W.-H., Thissen D. (1997). Local dependence indexes for item pairs using item response theory. Journal of Educational and Behavioral Statistics.

[b0100] Cheong, Y.F., Maxwell, L., & Yount, K.M. (nd). A practical guide for using item-response theory in scale evaluation of data from the project-level Women's Empowerment in Agriculture Index (pro-WEAI). International Food Policy Research Institute. Washington, D.C.

[b0105] Cheong Y.F., Crandall A., Yount K.M. (2017). Longitudinal measurement invariance of the women’s agency scale. Bulletin of Sociological Methodology/Bulletin de Méthodologie Sociologique.

[b0110] Crandall A., Cheong Y.F., Yount K.M. (2015). Validation of the general self-efficacy scale among Qatari young women. Eastern Mediterranean Health Journal.

[b0115] Deci E.L., Ryan R.M. (1985). Intrinsic motivation and self-determination in human behavior.

[b0120] Deci E.L., Ryan R.M., Kernis M.H. (1995). Human autonomy: The basis for true self-esteem. Efficacy, agency and self-esteem.

[b0125] Deci E.L., Ryan R.M. (2000). The “what” and “why” of goal pursuits: Human needs and the self determination of behavior. Psychological Inquiry.

[b0130] Dijkstra A.G. (2002). Revisiting UNDP's GDI and GEM: Towards an alternative. Social Indicators Research.

[b0135] Dijkstra A.G., Hanmer L.C. (2000). Measuring socio-economic gender inequality: Toward an alternative to the UNDP gender-related development index. Feminist Economics.

[b0140] Embretson S.E., Reise S. (2000). Item response theory for psychologists.

[b0145] Freire P. (1972). Pedagogy of the oppressed. 1968.

[b0150] Freire P. (2018). Pedagogy of the oppressed.

[b0155] Grootaert C., Narayan D., Jones V.N., Woolcock M. (2004). Measuring social capital: An integrated questionnaire.

[b0160] Hagtvet K.A., Sipos K. (2016). Creating short forms for construct measures: The role of exchangeable forms. Pedagogika.

[b0165] Hannan A., Heckert J., James-Hawkins L., Yount K.M. (2019). Development and validation of a nutrition and health module for the project-level Women’s Empowerment in Agriculture Index. Maternal and Child Nutrition.

[b0170] Hu L., Bentler P.M. (1999). Cutoff criteria for fit indexes in covariance structure analysis: Conventional criteria versus new alternatives. Structural Equation Modeling: A Multidisciplinary Journal.

[b0175] ICF International. (2011). Demographic and health surveys methodology – Questionnaires: Household, woman’s, and man’s. MEASURE DHS Phase III. Retrieved from Calverton, Maryland, USA: http://www.measuredhs.com/publications/publication-DHSQ6-DHS-Questionnaires-and-Manuals.cfm.

[b0180] James-Hawkins L., Peters C., VanderEnde K., Bardin L., Yount K.M. (2016). Women's agency and its relationship to current contraceptive use in low, lower-middle, and upper-middle income countries: A systematic review of the literature. Global Public Health.

[b0185] Kabeer N. (1999). Resources, agency, achievements: Reflections on the measurement of women's empowerment. Development and Change.

[b0190] Kabeer N. (2005). Gender equality and women's empowerment: A critical analysis of the third millennium development goal 1. Gender & Development.

[b0195] Kishor, S., & Subaiya, L. (2008). Understanding womens empowerment: a comparative analysis of Demographic and Health Surveys (DHS) data. Retrieved from Calverton, MD: https://dhsprogram.com/pubs/pdf/CR20/CR20.pdf.

[b0200] Klasen S. (2006). UNDP's gender-related measures: Some conceptual problems and possible solutions. Journal of Human Development.

[b0205] Komter A. (1989). Hidden power in marriage. Gender & Society.

[b0210] Kumar N., Raghunathan K., Arrieta A., Jilani A., Chakrabarti S., Menon P., Quisumbing A.R. (2019). Social networks, mobility, and political participation: The potential for women’s self-help groups to improve access and use of public entitlement schemes in India. World Development.

[b0215] Langsten R., Salem R. (2008). Two approaches to measuring women's work in developing countries: A comparison of survey data from Egypt. Population and Development Review.

[b0220] Lord F. (1980). Application of item response theory to practical testing problems.

[b0225] Lukes S. (1974). Power: A radical view.

[b0230] Mahmud S., Shah N.M., Becker S. (2012). Measurement of women’s empowerment in rural Bangladesh. World Development.

[b0235] Malapit H., Quisumbing A., Meinzen-Dick R., Seymour G., Martinez E.M., Heckert J., Gender Agriculture Assets Phase II Study Team (2019). Development of the project-level Women’s Empowerment in Agriculture Index (pro-WEAI). World Development.

[b0240] Malapit, H. J. L., Pinkstaff, C., Sproule, K., Kovarik, C., Quisumbing, A. R., & Meinzen-Dick, R. S. (2017). The Abbreviated Women's Empowerment in Agriculture Index (A-WEAI). IFPRI Discussion Paper 1647.

[b0245] Malhotra A., Schuler S.R. (2005). Women’s empowerment as a variable in international development. Measuring empowerment: Cross-disciplinary perspectives.

[b0250] Marsh H.W., Ellis L.A., Parada R.H., Richards G., Heubeck B.G. (2005). A short version of the self description questionnaire II: Operationalizing criteria for short-form evaluation with new applications of confirmatory factor analyses. Psychological Assessment.

[b0255] Mason K.O. (1986). The status of women: Conceptual and methodological issues in demographic studies. Paper presented at the Sociological forum.

[b0260] Mason K.O., Narayan D. (2005). Measuring women’s empowerment: Learning from cross-national research. Measuring empowerment: Cross-disciplinary perspectives.

[b0265] Mason K.O., Smith H.L. (2003). Women’s empowerment and social context: Results from five Asian countries.

[b0270] Meade A.W., Lautenschlager G.J. (2004). A comparison of item response theory and confirmatory factor analytic methodologies for establishing measurement equivalence/invariance. Organizational Research Methods.

[b0275] Meinzen-Dick, R. S., Rubin, D., Elias, M., Mulema, A. A., & Myers, E. (2019). Women’s empowerment in agriculture: Lessons from qualitative research. Retrieved from Washington, DC: http://ebrary.ifpri.org/cdm/ref/collection/p15738coll2/id/133060.

[b0280] Miedema S.S., Haardörfer R., Girard A.W., Yount K.M. (2018). Women’s empowerment in East Africa: Development of a cross-country comparable measure. World Development.

[b0285] Muthén L.K., Muthén B.O. (1998-2017). Mplus user's guide.

[b0290] O’Hara C., Clement F. (2018). Power as agency: A critical reflection on the measurement of women’s empowerment in the development sector. World Development.

[b0295] Pitt M.M., Khandker S.R., Cartwright J. (2006). Empowering women with micro finance: Evidence from Bangladesh. Economic Development and Cultural Change.

[b0300] Presser H., Sen G. (2000). Women's empowerment and demographic processes: Moving beyond.

[b0310] Richardson R.A. (2018). Measuring women's empowerment: A need for context and caution. The Lancet Global Health.

[b0315] Rowlands J. (1995). Empowerment examined. Development in Practice.

[b0320] Rowlands J. (1997). Questioning empowerment: Working with women in Honduras.

[b0325] Rowlands J., Afshar H. (1998). A word of the times, but what does it mean? Empowerment in the discourse and practice of development. Women and empowerment: Illustrations from the third world.

[b0330] Ryan R.M., Deci E.L. (2000). Self-determination theory and the facilitation of intrinsic motivation, social development, and well-being. American Psychologist.

[b0335] Samejima F. (1969). Estimation of latent ability using a response pattern of graded scores.

[b0340] Schmidt F.L., Le H., Ilies R. (2003). Beyond alpha: An empirical examination of the effects of different sources of measurement error on reliability estimates for measures of individual differences constructs. Psychological Methods.

[b0345] Scholz U., Doña B.G., Sud S., Schwarzer R. (2002). Is general self-efficacy a universal construct? Psychometric findings from 25 countries. European Journal of Psychological Assessment.

[b0350] Seymour G., Peterman A. (2018). Context and measurement: An analysis of the relationship between intrahousehold decision making and autonomy. World Development.

[b0355] Shavelson R.J., Webb N.M. (1981). Generalizability theory: 1973–1980. British Journal of Mathematical and Statistical Psychology.

[b0360] Sherer M., Maddux J.E., Mercandante B., Prentice-Dunn S., Jacobs B., Rogers R.W. (1982). The self-efficacy scale: Construction and validation. Psychological Reports.

[b0365] Smith G.T., McCarthy D.M., Anderson K.G. (2000). On the sins of short-form development. Psychological Assessment.

[b0370] Smith L.C. (2003). The importance of women's status for child nutrition in developing countries (Vol. 131).

[b0375] Stanton J.M., Sinar E.F., Balzer W.K., Smith P.C. (2002). Issues and strategies for reducing the length of self-report scales. Personnel Psychology.

[b0380] StataCorp (2017). Stata statistical software: Release 15.

[b0385] Stromquist N.P. (1995). The theoretical and practical bases for empowerment.

[b0390] Tay L., Meade A.W., Cao M. (2015). An overview and practical guide to IRT measurement equivalence analysis. Organizational Research Methods.

[b0395] Thorpe S., VanderEnde K., Peters C., Bardin L., Yount K.M. (2015). The influence of women's empowerment on child immunization coverage in low, lower-middle, and upper-middle income countries: A systematic review of the literature. Maternal and Child Health Journal.

[b0400] Toland M.D. (2014). Practical guide to conducting an item response theory analysis. The Journal of Early Adolescence.

[b0405] United Nations Development Programme [UNDP]. (2018). Human development indices and indicators 2018 statistical update. Retrieved from New York.

[b0410] United Nations General Assembly. (2015). Transforming our World: The 2030 agenda for sustainable development (A/RES/70/1). Retrieved from New York.

[b0415] Weber M., Gerth H., Mills C.W. (1946). Class, status, party. From Max Weber: Essays in sociology.

[b0420] World Economic Forum. (2018). The global gender gap report 2018. Retrieved from Geneva.

[b0425] Yount K.M., Crandall A., Cheong Y.F. (2018). Women's age at first marriage and longterm economic empowerment in Egypt. World Development.

[b0430] Yount, K. M., James-Hawkins, L., & Abdul-Rahim, H. (nd). The Women's Agency in Pregnancy Scale (WAPS): Development and validation in a cross-sectional study of Qatari and Non-Qatari Women. 10.1186/s12884-020-03205-2 PMC7466495 32873247

[b0435] Yount K.M., VanderEnde K., Zureick-Brown S., Anh H.T., Schuler S.R., Minh T.H. (2014). Measuring attitudes about intimate partner violence against women: The ATT-IPV scale. Demography.

[b0440] Yount K.M., VanderEnde K., Zureick-Brown S., Hoang T.A., Schuler S.R., Minh T.H. (2014). Measuring attitudes about intimate partner violence against women: The ATT-IPV scale. Demography.

[b0445] Yount K.M., VanderEnde K.E., Dodell S., Cheong Y.F. (2016). Measurement of women’s agency in Egypt: A national validation study. Social indicators research.

[b0450] Yount K.M., Zureick-Brown S., Salem R. (2014). Intimate partner violence and women's economic and non-economic activities in Minya, Egypt. Demography.

[b0455] Yu, C.-Y. (2002). Evaluating cutoff criteria of model fit indices for latent variable models with binary and continuous outcomes (Doctoral dissertation). Los Angeles, CA.

