# Supplementary material for: Measurement properties of the project-level Women's Empowerment in Agriculture Index
Source: World Dev. 2019 Dec;124:104639. doi: 10.1016/j.worlddev.2019.104639 (PMC6876673; doi:10.1016/j.worlddev.2019.104639)
Supplement: Supplementary data 1 [file mmc1.pdf]

Appendix Table 1

PROJECT-LEVEL WOMEN'S EMPOWERMENT IN AGRICULTURE INDEX
PILOT VERSION
MAY 2018

These survey modules are a DRAFT version of the project-level Women's Empowerment in Agriculture Index (pro-WEAI). Optional questions and modules are designated in purple text. The survey questions, format, and required portions are subject to change as the pro-WEAI continues to develop. Updated survey modules may be available from the pro-WEAI team.

Pro-WEAI is a survey-based index for measuring empowerment, agency, and inclusion of women in the agriculture sector. It is being developed jointly by the International Food Policy Research Institute (IFPRI), the Oxford Policy and Human Development Initiative (OHPI), and thirteen partner projects in the portfolio of the Gender, Agriculture, and Assets Project, Phase 2 (GAAP2). The tool helps agricultural development projects assess women's empowerment in a project setting, diagnose areas of women's disempowerment, design strategies to address deficiencies, and monitor project outcomes. Pro-WEAI is an adaptation of the Women's Empowerment in Agriculture Index (WEAI), originally developed in 2012 by IFPRI, the United States Agency for International Development (USAID), and OPHI.

For more information about pro-WEAI, please visit weai.ifpri.info or email Hazel Malapit at h.malapit@cgiar.org.

DRAFT

DRAFT

MODULE G. WOMEN'S EMPOWERMENT IN AGRICULTURE INDEX – Pilot Pro-WEAI Version

Note to survey designers: The information in module G1 can be captured in different ways; however, there must be a way to: (a) identify the proper individual within the household to be asked the survey, (b) link this individual from the module to the household roster, (c) code the outcome of the interview, especially if the individual is not available, to distinguish this from missing data, and (d) record who else in the household was present during the interview. This instrument must be adapted for country context including adding relevant examples and translations into local languages when appropriate.

Note to enumerators: *This questionnaire should be administered separately to the primary and secondary respondents identified in the household roster of the household level questionnaire. You should complete this coversheet for each individual identified in the "selection section" even if the individual is not available to be interviewed for reporting purposes. For some surveys (such as those focusing on nutrition outcomes), the female respondent may be the beneficiary woman or mother or primary caregiver of the index child (also the respondent for the pro-WEAI nutrition module). Please make sure that she is also the person interviewed for this questionnaire and that the male respondent is her spouse/partner (if applicable).*

Please double-check to ensure:

- You have completed the roster section of the household questionnaire to identify the correct primary and/or secondary respondent(s);
- You have noted the household ID and individual ID correctly for the person you are about to interview;
- You have gained informed consent from the individual in the household questionnaire;
- You have sought to interview the individual in private or where other members of the household cannot overhear or contribute answers.
- Do not attempt to make responses between the primary and secondary respondents the same—it is okay for them to be different.

MODULE G1. INDIVIDUAL IDENTIFICATION

**G1.01. HOUSEHOLD IDENTIFICATION:**	|__|__|__|__|__|__|	**G1.04 TYPE OF HOUSEHOLD**	MALE AND FEMALE ADULT....1<br>FEMALE ADULT ONLY....2
**G1.02. NAME OF RESPONDENT CURRENTLY BEING INTERVIEWED (ID CODE FROM ROSTER IN SECTION B HOUSEHOLD ROSTER):**	|__|__|	**G1.05. OUTCOME OF INTERVIEW:**<br><br>**CIRCLE ONE**	COMPLETED....1<br>HOUSEHOLD MEMBER TOO ILL TO RESPOND/COGNITIVELY IMPAIRED...2<br>RESPONDENT NOT AT HOME/TEMPORARILY UNAVAILABLE....3<br>RESPONDENT NOT AT HOME/EXTENDED ABSENCE....4<br>REFUSED....5<br>COULD NOT LOCATE....6
**SURNAME, OTHER NAME:** ______________________________			
**G1.03. SEX OF RESPONDENT:**	MALE....1<br>FEMALE....2	**G1.06. ABILITY TO BE INTERVIEWED ALONE:**<br><br>**CIRCLE ONE**	ALONE....1<br>WITH ADULT FEMALES PRESENT....2<br>WITH ADULT MALES PRESENT....3<br>WITH ADULTS OF BOTH SEX PRESENT....4<br>WITH CHILDREN PRESENT....5<br>WITH ADULTS OF BOTH SEX AND CHILDREN PRESENT....6

HOUSEHOLD IDENTIFICATION (IN DATA FILE, EACH SUB-MODULE (G2-G8) MUST BE LINKED WITH A HH AND RESPONDENT ID)

HOUSEHOLD ID						
RESPONDENT ID						

MODULE G2: ROLE IN HOUSEHOLD DECISION-MAKING AROUND PRODUCTION AND INCOME

	Now I'd like to ask you some questions about your participation in certain types of work activities and on making decisions on various aspects of household life.	Did you [NAME] participate in [ACTIVITY] in the past 12 months (that is, during the last [one/two] cropping seasons), from [PRESENT MONTH] last year to [PRESENT MONTH] this year?	When decisions are made regarding [ACTIVITY], who is it that normally takes the decision?<br><br>**ENTER UP TO THREE (3) MEMBER IDs**<br><br>**IF RESPONSE IS <u>MEMBER ID (SELF) ONLY</u> → *G2.05***<br><br>**OTHER CODES:**<br>NON-HH MEMBER.......94<br>NOT APPLICABLE.......98 → ***NEXT ACTIVITY***			How much input did you have in making decisions about [ACTIVITY]?<br><br>**USE CODE G2↓**	To what extent do you feel you can participate in decisions regarding [ACTIVITY] if you want(ed) to?<br><br>**CIRCLE <u>ONE</u>**	To what extent are you able to access information that you feel is important for making informed decisions regarding [ACTIVITY]?<br><br>**CIRCLE <u>ONE</u>**	How much input did you have in decisions about how much of the outputs of [ACTIVITY] to keep for consumption at home rather than selling?<br><br>**USE CODE G2↓**	How much input did you have in decisions about how to use income generated from [ACTIVITY]?<br><br>**USE CODE G2↓**
	**ACTIVITY**	**G2.01**	**G2.02**			**G2.03**	**G2.04**	**G2.05**	**G2.06**	**G2.07**
			**ID #1**	**ID #2**	**ID #3**					
**A**	Staple grain farming and processing of the harvest: grains that are grown primarily for food consumption (rice, maize, wheat, millet)	YES......1<br>NO.......2 → ***ACTIVITY B***					NOT AT ALL.............1<br>SMALL EXTENT........2<br>MEDIUM EXTENT......3<br>TO A HIGH EXTENT...4	NOT AT ALL.............1<br>SMALL EXTENT........2<br>MEDIUM EXTENT......3<br>TO A HIGH EXTENT...4		
**B**	Horticultural (gardens) or high value crop farming and processing of the harvest	YES......1<br>NO.......2 → ***ACTIVITY C***					NOT AT ALL.............1<br>SMALL EXTENT........2<br>MEDIUM EXTENT......3<br>TO A HIGH EXTENT...4	NOT AT ALL.............1<br>SMALL EXTENT........2<br>MEDIUM EXTENT......3<br>TO A HIGH EXTENT...4		
**C**	Large livestock raising (cattle, buffaloes) and processing of milk and/or meat	YES......1<br>NO.......2 → ***ACTIVITY D***					NOT AT ALL.............1<br>SMALL EXTENT........2<br>MEDIUM EXTENT......3<br>TO A HIGH EXTENT...4	NOT AT ALL.............1<br>SMALL EXTENT........2<br>MEDIUM EXTENT......3<br>TO A HIGH EXTENT...4		
**D**	Small livestock raising (sheep, goats, pigs) and processing of milk and/or meat	YES......1<br>NO.......2 → ***ACTIVITY E***					NOT AT ALL.............1<br>SMALL EXTENT........2<br>MEDIUM EXTENT......3<br>TO A HIGH EXTENT...4	NOT AT ALL.............1<br>SMALL EXTENT........2<br>MEDIUM EXTENT......3<br>TO A HIGH EXTENT...4		
**E**	Poultry and other small animals raising (chickens, ducks, turkeys) and processing of eggs and/or meat	YES......1<br>NO.......2 → ***ACTIVITY F***					NOT AT ALL.............1<br>SMALL EXTENT........2<br>MEDIUM EXTENT......3<br>TO A HIGH EXTENT...4	NOT AT ALL.............1<br>SMALL EXTENT........2<br>MEDIUM EXTENT......3<br>TO A HIGH EXTENT...4		

DRAFT

CODE G2	
LITTLE TO NO INPUT IN DECISIONS	1
INPUT INTO SOME DECISIONS	2
INPUT INTO MOST OR ALL DECISIONS	3
NOT APPICABLE / NO DECISION MADE	98

DRAFT

		Did you [NAME] participate in [ACTIVITY] in the past 12 months (that is, during the last [one/two] cropping seasons), from [PRESENT MONTH] last year to [PRESENT MONTH] this year?	When decisions are made regarding [ACTIVITY], who is it that normally takes the decision?<br>**ENTER UP TO THREE (3) MEMBER IDs**<br><br>**IF RESPONSE IS <u>MEMBER ID (SELF) ONLY</u> → *G2.05***<br><br>**OTHER CODES:**<br>NON-HH MEMBER.......94<br>NOT APPLICABLE.......98 → ***NEXT ACTIVITY***			How much input did you have in making decisions about [ACTIVITY]?<br><br>**USE CODE G2↓**	To what extent do you feel you can participate in decisions regarding [ACTIVITY] if you want(ed) to?<br><br>**CIRCLE <u>ONE</u>**	To what extent are you able to access information that you feel is important for making informed decisions regarding [ACTIVITY]?<br><br>**CIRCLE <u>ONE</u>**	How much input did you have in decisions about how much of the outputs of [ACTIVITY] to keep for consumption at home rather than selling?<br><br>**USE CODE G2↓**	How much input did you have in decisions about how to use income generated from [ACTIVITY]?<br><br>**USE CODE G2↓**
**ACTIVITY**		**G2.01**	**G2.02**			**G2.03**	**G2.04**	**G2.05**	**G2.06**	**G2.07**
			**ID #1**	**ID #2**	**ID #3**					
**F**	Fishpond culture	YES......1<br>NO.......2 → ***ACTIVITY G***					NOT AT ALL.............1<br>SMALL EXTENT........2<br>MEDIUM EXTENT......3<br>TO A HIGH EXTENT...4	NOT AT ALL.............1<br>SMALL EXTENT........2<br>MEDIUM EXTENT......3<br>TO A HIGH EXTENT...4		
**G**	Non-farm economic activities (running a small business, self-employment, buy-and-sell)	YES......1<br>NO.......2 → ***ACTIVITY H***					NOT AT ALL.............1<br>SMALL EXTENT........2<br>MEDIUM EXTENT......3<br>TO A HIGH EXTENT...4	NOT AT ALL.............1<br>SMALL EXTENT........2<br>MEDIUM EXTENT......3<br>TO A HIGH EXTENT...4		
**H**	Wage and salary employment (work that is paid for in cash or in-kind, including both agriculture and other wage work)	YES......1<br>NO.......2 → ***ACTIVITY I***					NOT AT ALL.............1<br>SMALL EXTENT........2<br>MEDIUM EXTENT......3<br>TO A HIGH EXTENT...4	NOT AT ALL.............1<br>SMALL EXTENT........2<br>MEDIUM EXTENT......3<br>TO A HIGH EXTENT...4		
**I**	Large, occasional household purchases (bicycles, land, transport vehicles)						NOT AT ALL.............1<br>SMALL EXTENT........2<br>MEDIUM EXTENT......3<br>TO A HIGH EXTENT...4	NOT AT ALL.............1<br>SMALL EXTENT........2<br>MEDIUM EXTENT......3<br>TO A HIGH EXTENT...4		
**J**	Routine household purchases (food for daily consumption or other household needs)						NOT AT ALL.............1<br>SMALL EXTENT........2<br>MEDIUM EXTENT......3<br>TO A HIGH EXTENT...4	NOT AT ALL.............1<br>SMALL EXTENT........2<br>MEDIUM EXTENT......3<br>TO A HIGH EXTENT...4		

DRAFT

CODE G2	
LITTLE TO NO INPUT IN DECISIONS..................................	1
INPUT INTO SOME DECISIONS	2
INPUT INTO MOST OR ALL DECISIONS.............................	3
NOT APPLICABLE / NO DECISION MADE	98

DRAFT

HOUSEHOLD ID						
RESPONDENT ID						

MODULE G3(A): ACCESS TO PRODUCTIVE CAPITAL

DRAFT

Now I'd like to ask you specifically about your household's land.

QUESTION		RESPONSE		
**G3.01.** Does anyone in your household currently own or cultivate land?		YES........1<br>NO.........2 → ***G3.06, ITEM A***		
**G3.02.** Who generally makes decisions about what to plant on this land?	**ENTER UP TO THREE (3) MEMBER IDs**<br><br>**OTHER CODES:**<br>NON-HH MEMBER............................94<br>NOT APPLICABLE.............................98	**ID #1**	**ID #2**	**ID #3**
**G3.03.** Do you [NAME] solely or jointly cultivate any land?	**CIRCLE ONE**	YES, SOLELY .... 1<br>YES, JOINTLY........ 2<br>YES, SOLELY AND JOINTLY........ 3<br>NO 4		
**G3.04.** Who generally makes decisions about what to plant on the land that you yourself cultivate?	**ENTER UP TO THREE (3) MEMBER IDs**<br><br>**OTHER CODES:**<br>NON-HH MEMBER............................94<br>NOT APPLICABLE.............................98	**ID #1**	**ID #2**	**ID #3**
**G3.05.** Do you own any of the land owned or cultivated by your household?	**CIRCLE ONE**	YES, SOLELY .... 1<br>YES, JOINTLY........ 2<br>YES, SOLELY AND JOINTLY........ 3<br>NO 4		

Now I'd like to ask you about a number of items that could be used to generate income.		Does anyone in your household currently have any [ITEM]?	Do you [NAME] own any [ITEM]?<br>**CIRCLE ONE**
**ITEM**		**G3.06**	**G3.07**
**A**	Large livestock (cattle, buffaloes)	YES........1<br>NO.........2 → ***ITEM B***	YES, SOLELY....1<br>YES, JOINTLY....2<br>YES, SOLELY AND JOINTLY....3<br>NO....4
**B**	Small livestock (sheep, goats, pigs)	YES........1<br>NO.........2 → ***ITEM C***	YES, SOLELY....1<br>YES, JOINTLY....2<br>YES, SOLELY AND JOINTLY....3<br>NO....4
**C**	Poultry and other small animals (chickens, ducks, turkeys)	YES........1<br>NO.........2 → ***ITEM D***	YES, SOLELY....1<br>YES, JOINTLY....2<br>YES, SOLELY AND JOINTLY....3<br>NO....4
**D**	Fish pond or fishing equipment	YES........1<br>NO.........2 → ***ITEM E***	YES, SOLELY....1<br>YES, JOINTLY....2<br>YES, SOLELY AND JOINTLY....3<br>NO....4
**E**	Non-mechanized farm equipment (hand tools, animal-drawn plough)	YES........1<br>NO.........2 → ***ITEM F***	YES, SOLELY....1<br>YES, JOINTLY....2<br>YES, SOLELY AND JOINTLY....3<br>NO....4
**F**	Mechanized farm equipment (tractor-plough, power tiller, treadle pump)	YES........1<br>NO.........2 → ***ITEM G***	YES, SOLELY....1<br>YES, JOINTLY....2<br>YES, SOLELY AND JOINTLY....3<br>NO....4
**G**	Non-farm business equipment (solar panels used for recharging, sewing machine, brewing equipment, fryers)	YES........1<br>NO.........2 → ***ITEM H***	YES, SOLELY....1<br>YES, JOINTLY....2<br>YES, SOLELY AND JOINTLY....3<br>NO....4
**H**	House or building	YES........1<br>NO.........2 → ***ITEM I***	YES, SOLELY....1<br>YES, JOINTLY....2<br>YES, SOLELY AND JOINTLY....3<br>NO....4
**I**	Large consumer durables (refrigerator, TV, sofa)	YES........1<br>NO.........2 → ***ITEM J***	YES, SOLELY....1<br>YES, JOINTLY....2<br>YES, SOLELY AND JOINTLY....3<br>NO....4

DRAFT

		Does anyone in your household currently own any [ITEM]?	Do you [NAME] own any [ITEM]?<br><br>**CIRCLE ONE**
**ITEM**		**G3.06**	**G3.07**
**J**	Small consumer durables (radio, cookware)	YES........1<br>NO.........2 → ***ITEM K***	YES, SOLELY .... 1<br>YES, JOINTLY........ 2<br>YES, SOLELY AND JOINTLY........ 3<br>NO........ 4
**K**	Cell phone	YES........1<br>NO.........2 → ***ITEM L***	YES, SOLELY .... 1<br>YES, JOINTLY........ 2<br>YES, SOLELY AND JOINTLY........ 3<br>NO........ 4
**L**	Other land not used for agricultural purposes (pieces/plots, residential or commercial land)	YES........1<br>NO.........2 → ***ITEM M***	YES, SOLELY .... 1<br>YES, JOINTLY........ 2<br>YES, SOLELY AND JOINTLY........ 3<br>NO........ 4
**M**	Means of transportation (bicycle, motorcycle, car)	YES........1<br>NO.........2 → ***MODULE G3(B)***	YES, SOLELY .... 1<br>YES, JOINTLY........ 2<br>YES, SOLELY AND JOINTLY........ 3<br>NO........ 4

DRAFT

MODULE G3(B): ACCESS TO FINANCIAL SERVICES

Next I'd like to ask about your household's experience with borrowing money or other items (in-kind) in the past 12 months.		Would you or anyone in your household be able to take a loan or borrow cash/in-kind from [SOURCE] if you wanted to?	Has anyone in your household taken any loans or borrowed cash/in-kind from [SOURCE] in the past 12 months?<br><br>**CIRCLE <u>ONE</u>**	Who made the decision to borrow from [SOURCE] most of the time?<br><br>**ENTER UP TO THREE (3) MEMBER IDs**<br><br>**OTHER CODES:**<br>NON-HH MEMBER.......94<br>NOT APPLICABLE.......98			Who makes the decision about what to do with the money or item borrowed from [SOURCE] most of the time?<br><br>**ENTER UP TO THREE (3) MEMBER IDs**<br><br>**OTHER CODES:**<br>NON-HH MEMBER.......94<br>NOT APPLICABLE.......98			Who is responsible for repaying the money or item borrowed from [SOURCE]?<br><br>**ENTER UP TO THREE (3) MEMBER IDs**<br><br>**OTHER CODES:**<br>NON-HH MEMBER.......94<br>NOT APPLICABLE.......98		
**LENDING SOURCES**		**G3.08**	**G3.09**	**G3.10**			**G3.11**			**G3.12**		
				**ID #1**	**ID #2**	**ID #3**	**ID #1**	**ID #2**	**ID #3**	**ID #1**	**ID #2**	**ID #3**
**A**	Non-governmental organization (NGO)	YES..........1<br>NO...........2 → ***SOURCE B***<br>MAYBE.....3	YES, CASH............................1<br>YES, IN-KIND ....2<br>YES, CASH AND IN-KIND ....3<br>NO...4<br>DON'T KNOW.....................97<br>(4, 97) → ***SOURCE B***									
**B**	Formal lender (bank/financial institution)	YES..........1<br>NO...........2 → ***SOURCE C***<br>MAYBE.....3	YES, CASH............................1<br>YES, IN-KIND ....2<br>YES, CASH AND IN-KIND ....3<br>NO...4<br>DON'T KNOW.....................97<br>(4, 97) → ***SOURCE C***									
**C**	Informal lender	YES..........1<br>NO...........2 → ***SOURCE D***<br>MAYBE.....3	YES, CASH............................1<br>YES, IN-KIND ....2<br>YES, CASH AND IN-KIND ....3<br>NO...4<br>DON'T KNOW.....................97<br>(4, 97) → ***SOURCE D***									
**D**	Friends or relatives	YES..........1<br>NO...........2 → ***SOURCE E***<br>MAYBE.....3	YES, CASH............................1<br>YES, IN-KIND ....2<br>YES, CASH AND IN-KIND ....3<br>NO...4<br>DON'T KNOW.....................97<br>(4, 97) → ***SOURCE E***									
**E**	Group based micro-finance or lending including VSLAs / SACCOs	YES..........1<br>NO...........2 → ***SOURCE F***<br>MAYBE.....3	YES, CASH............................1<br>YES, IN-KIND ....2<br>YES, CASH AND IN-KIND ....3<br>NO...4<br>DON'T KNOW.....................97<br>(4, 97) → ***SOURCE F***									
**F**	Informal credit / savings groups (.e.g., merry-go-rounds, tontines, funeral societies, etc.)	YES..........1<br>NO...........2 → ***G3.13***<br>MAYBE.....3	YES, CASH............................1<br>YES, IN-KIND ....2<br>YES, CASH AND IN-KIND ....3<br>NO...4<br>DON'T KNOW.....................97<br>(4, 97) → ***G3.13***									

DRAFT

**G3.13**	An account can be used to save money, to make or receive payments, or to receive wages or financial help. Do you, either by yourself or together with someone else, currently have an account at any of the following places: a bank or other formal institution (e.g., post office)?	YES......1<br>NO ......2<br>DON'T KNOW 97

DRAFT

HOUSEHOLD ID						
RESPONDENT ID		

MODULE G4: TIME ALLOCATION

G4.01: PLEASE RECORD A LOG OF THE ACTIVITIES FOR THE INDIVIDUAL IN THE LAST COMPLETE 24 HOURS (STARTING YESTERDAY MORNING AT 4 AM, FINISHING 3:59 AM OF THE CURRENT DAY). THE TIME INTERVALS ARE MARKED IN 15 MIN INTERVALS. MARK ONE ACTIVITY FOR EACH TIME PERIOD BY ENTERING THE CORRESPONDING ACTIVITY CODE IN THE BOX.

G4.02: CHECK THE BOX BELOW IF THE RESPONDENT WAS CARING FOR CHILDREN WHILE PERFORMING EACH ACTIVITY.

Now I'd like to ask you about how you spent your time during the past 24 hours. We'll begin from yesterday morning, and continue through to this morning. This will be a detailed accounting. I'm interested in everything you did (i.e. resting, eating, personal care, work inside and outside the home, caring for children, cooking, shopping, socializing, etc.), even if it didn't take you much time. I'm particularly interested in agricultural activities such as farming, gardening, and livestock raising whether in the field or on the homestead. I'm also interested in how much time you spent caring for children, especially if it happened while you did some other activity (e.g., collecting water while carrying a child or cooking while watching after a sleeping child).

		Night		Morning	Day								
		4:00	5:00	6:00	7:00	8:00	9:00	10:00	11:00	12:00	13:00	14:00	15:00
**G4.01** Activity **(WRITE ACTIVITY CODE)**													
**G4.02** Did you also care for children?	YES..........CHECK BOX<br>NO.........LEAVE BLANK	☐ ☐ ☐ ☐	☐ ☐ ☐ ☐	☐ ☐ ☐ ☐	☐ ☐ ☐ ☐	☐ ☐ ☐ ☐	☐ ☐ ☐ ☐	☐ ☐ ☐ ☐	☐ ☐ ☐ ☐	☐ ☐ ☐ ☐	☐ ☐ ☐ ☐	☐ ☐ ☐ ☐	☐ ☐ ☐ ☐

		Day		Evening	Night								
		16:00	17:00	18:00	19:00	20:00	21:00	22:00	23:00	24:00	1:00	2:00	3:00
**G4.01** Activity **(WRITE ACTIVITY CODE)**													
**G4.02** Did you also care for children?	YES..........CHECK BOX<br>NO.........LEAVE BLANK	☐ ☐ ☐ ☐	☐ ☐ ☐ ☐	☐ ☐ ☐ ☐	☐ ☐ ☐ ☐	☐ ☐ ☐ ☐	☐ ☐ ☐ ☐	☐ ☐ ☐ ☐	☐ ☐ ☐ ☐	☐ ☐ ☐ ☐	☐ ☐ ☐ ☐	☐ ☐ ☐ ☐	☐ ☐ ☐ ☐

**ACTIVITY CODES FOR G4.01**			
A……………..Sleeping and resting<br>B......................Eating and drinking<br>C…………...……..Personal care<br>D…………School (incl. homework)<br>E……………..…Work as employed<br>F……………..…Own business work<br>G……………...Staple grain farming	H………..Horticultural (gardens) or high value crop farming<br>I……………….…..Large livestock raising (cattle, buffaloes)<br>J……………...….Small livestock raising (sheep, goats, pigs)<br>K..............................Poultry and other small animals raising (chickens, ducks, turkeys)<br>L…………………………………………….…Fishpond culture<br>M………………………Commuting (to/from work or school)	N……….Shopping / getting service (incl. health services)<br>O……………………...…..Weaving / sewing / textile care<br>P………………………………………………….…..Cooking<br>Q…..………..Domestic work (incl. fetching water and fuel)<br>R…………………………………………..Caring for children<br>S……………………...…..Caring for adults (sick, elderly)<br>T……………………..…..Traveling (not for work or school)	U………………………..Exercising<br>V……Social activities and hobbies<br>W……..………Religious activities<br>X…………………..….Other (specify)

DRAFT

**G4.03.** In the last 24 hours did you work (at home or outside of the home including chores or other domestic activities) less than usual, about the same as usual, or more than usual?	**FOR <u>FEMALES</u> ONLY: DOES RESPONDENT HAVE A CHILD UNDER 5 YEARS OLD?**	**G4.04.** If you wanted to do something (livelihood-related, training-related, self-care) and could not take your child with you, is there someone who could care for your child in your absence?	**G4.05.** Who?<br><br>**ENTER UP TO THREE (3) MEMBER IDs**	**ID #1**	**ID #2**	**ID #3**
LESS THAN USUAL……………………………1<br>ABOUT THE SAME AS USUAL…………..2<br>MORE THAN USUAL………………………3<br><br>**IF RESPONDENT IS <u>MALE</u> → MODULE G5**	YES………1 → G4.04<br>NO………..2 → MODULE G5	YES………1 → G4.05<br>NO………..2 → MODULE G5	**OTHER CODES:**<br>NON-HH MEMBER…….94<br>NOT APPLICABLE…….98			

DRAFT

HOUSEHOLD ID						
RESPONDENT ID		

MODULE G5: GROUP MEMBERSHIP

DRAFT

Now I'm going to ask you about groups in the community. These can be either formal or informal and customary groups.		Is there a [GROUP] in your community?		Is this group composed of all male or female or mixed-sex members?	Are you an active member of this [GROUP]?	To what extent do you feel like you can influence decisions in this [GROUP]?	To what extent does this [GROUP] influence life in the community beyond the group activities?
**GROUP CATEGORIES**		**G5.01**		**G5.02**	**G5.03**	**G5.04**	**G5.05**
**A**	Agricultural / livestock / fisheries producer's group (including marketing groups)	YES............................1<br>NO ....2<br>DON'T KNOW 97	***GROUP B***	ALL MALE..........................1<br>ALL FEMALE......................2<br>MIXED SEX.........................3<br>DON'T KNOW...................97	YES......1<br>NO........2 → ***GROUP B***	NOT AT ALL........................1<br>SMALL EXTENT..................2<br>MEDIUM EXTENT................3<br>HIGH EXTENT.....................4	NOT AT ALL........................1<br>SMALL EXTENT..................2<br>MEDIUM EXTENT................3<br>HIGH EXTENT.....................4
**B**	Water users' group	YES............................1<br>NO ....2<br>DON'T KNOW 97	***GROUP C***	ALL MALE..........................1<br>ALL FEMALE......................2<br>MIXED SEX.........................3<br>DON'T KNOW...................97	YES......1<br>NO........2 → ***GROUP C***	NOT AT ALL........................1<br>SMALL EXTENT..................2<br>MEDIUM EXTENT................3<br>HIGH EXTENT.....................4	NOT AT ALL........................1<br>SMALL EXTENT..................2<br>MEDIUM EXTENT................3<br>HIGH EXTENT.....................4
**C**	Forest users' group	YES............................1<br>NO ....2<br>DON'T KNOW 97	***GROUP D***	ALL MALE..........................1<br>ALL FEMALE......................2<br>MIXED SEX.........................3<br>DON'T KNOW...................97	YES......1<br>NO........2 → ***GROUP D***	NOT AT ALL........................1<br>SMALL EXTENT..................2<br>MEDIUM EXTENT................3<br>HIGH EXTENT.....................4	NOT AT ALL........................1<br>SMALL EXTENT..................2<br>MEDIUM EXTENT................3<br>HIGH EXTENT.....................4
**D**	Credit or microfinance group (including SACCOs / merry-go-rounds / VSLAs)	YES............................1<br>NO ....2<br>DON'T KNOW 97	***GROUP E***	ALL MALE..........................1<br>ALL FEMALE......................2<br>MIXED SEX.........................3<br>DON'T KNOW...................97	YES......1<br>NO........2 → ***GROUP E***	NOT AT ALL........................1<br>SMALL EXTENT..................2<br>MEDIUM EXTENT................3<br>HIGH EXTENT.....................4	NOT AT ALL........................1<br>SMALL EXTENT..................2<br>MEDIUM EXTENT................3<br>HIGH EXTENT.....................4
**E**	Mutual help or insurance group (including burial societies)	YES............................1<br>NO ....2<br>DON'T KNOW 97	***GROUP F***	ALL MALE..........................1<br>ALL FEMALE......................2<br>MIXED SEX.........................3<br>DON'T KNOW...................97	YES......1<br>NO........2 → ***GROUP F***	NOT AT ALL........................1<br>SMALL EXTENT..................2<br>MEDIUM EXTENT................3<br>HIGH EXTENT.....................4	NOT AT ALL........................1<br>SMALL EXTENT..................2<br>MEDIUM EXTENT................3<br>HIGH EXTENT.....................4
**F**	Trade and business association group	YES............................1<br>NO ....2<br>DON'T KNOW 97	***GROUP G***	ALL MALE..........................1<br>ALL FEMALE......................2<br>MIXED SEX.........................3<br>DON'T KNOW...................97	YES......1<br>NO........2 → ***GROUP G***	NOT AT ALL........................1<br>SMALL EXTENT..................2<br>MEDIUM EXTENT................3<br>HIGH EXTENT.....................4	NOT AT ALL........................1<br>SMALL EXTENT..................2<br>MEDIUM EXTENT................3<br>HIGH EXTENT.....................4
**G**	Civic group (improving community) or charitable group (helping others)	YES............................1<br>NO ....2<br>DON'T KNOW 97	***GROUP H***	ALL MALE..........................1<br>ALL FEMALE......................2<br>MIXED SEX.........................3<br>DON'T KNOW...................97	YES......1<br>NO........2 → ***GROUP H***	NOT AT ALL........................1<br>SMALL EXTENT..................2<br>MEDIUM EXTENT................3<br>HIGH EXTENT.....................4	NOT AT ALL........................1<br>SMALL EXTENT..................2<br>MEDIUM EXTENT................3<br>HIGH EXTENT.....................4

**H**	Religious group	YES............................ 1<br>NO .... 2 → ***GROUP I***<br>DON'T KNOW .... 97 → ***GROUP I***	ALL MALE............................1<br>ALL FEMALE.......................2<br>MIXED SEX..........................3<br>DON'T KNOW....................97	YES.......1<br>NO.........2 → ***GROUP I***	NOT AT ALL..........................1<br>SMALL EXTENT...................2<br>MEDIUM EXTENT.................3<br>HIGH EXTENT......................4	NOT AT ALL..........................1<br>SMALL EXTENT...................2<br>MEDIUM EXTENT.................3<br>HIGH EXTENT......................4
**I**	Other (specify): ________________________	YES............................ 1<br>NO .... 2 → ***MODULE G6***<br>DON'T KNOW .... 97 → ***MODULE G6***	ALL MALE............................1<br>ALL FEMALE.......................2<br>MIXED SEX..........................3<br>DON'T KNOW....................97	YES.......1<br>NO.........2 → ***MODULE G6***	NOT AT ALL..........................1<br>SMALL EXTENT...................2<br>MEDIUM EXTENT.................3<br>HIGH EXTENT......................4	NOT AT ALL..........................1<br>SMALL EXTENT...................2<br>MEDIUM EXTENT.................3<br>HIGH EXTENT......................4

DRAFT

HOUSEHOLD ID						
RESPONDENT ID		

MODULE G6. PHYSICAL MOBILITY

QUESTION	RESPONSE<br><br>FOR G6.01 - G6.06: USE CODE G6↓
**G6.01** How often do you visit an urban center?	
**G6.02** How often do you go to the market / haat / bazaar?	
**G6.03** How often do you go to visit family or relatives?	
**G6.04** How often do you go to visit a friend / neighbor's house?	
**G6.05** How often do you go to the hospital / clinic / doctor (seek health service)?	
**G6.06** How often do you go to a public village gathering / community meeting / training for NGO or programs?	
**G6.07.** In the last 12 months, how many times have you been away from home for one or more nights (in other words, sleeping somewhere else for the night)?	
**G6.08.** In the last 12 months, have you been away from home for more than one month at a time?	YES...1<br>NO..<br>2<br><br>***IF RESPONDENT IS MALE →MODULE G7***

CODE G6	
EVERYDAY	1
EVERY WEEK AT LEAST ONCE	2
EVERY 2 WEEKS AT LEAST ONCE	3
EVERY MONTH AT LEAST ONCE	4
LESS THAN ONCE A MONTH	5
NEVER	6

DRAFT

REMAINDER OF MODULE (G6.09-G6.08) SHOULD ONLY BE ASKED IF RESPONDENT IS FEMALE

Now I'd like to ask you some questions about different places you might visit.		Who usually decides whether you can go to [PLACE]?<br><br>**ENTER UP TO THREE (3) MEMBER IDs**<br><br>**IF RESPONSE IS MEMBER ID (SELF) ONLY → NEXT PLACE**<br><br>**OTHER CODES:**<br>NON-HH MEMBER.......94<br>NOT APPLICABLE.......98			Does your husband/partner or other household member object to you going alone to [PLACE]?	Under what circumstances would this person NOT object to your going to [PLACE] alone?<br><br>**CIRCLE ALL APPLICABLE**	Do these objections prevent you from going alone to [PLACE]?
**PLACE**		**G6.09**			**G6.10**	**G6.11**	**G6.12**
		**ID #1**	**ID #2**	**ID #3**			
**A**	Urban center				YES......1<br>NO........2 → ***PLACE B***	IF I HAVE COMPANY (RELATIVES, CHILDREN)...............................1<br>IF I CAN ARRANGE MY OWN EXPENSES (FOR TRANSPORT)............2<br>IF I FOLLOW PURDAH / DRESS ACCEPTABLY...............................3<br>OTHER (SPECIFY)..4<br>UNDER NO CIRCUMSTANCES WOULD I BE ALLOWED TO GO...........5 → ***PLACE B***	YES......1<br>NO........2
**B**	Market / haat / bazaar				YES......1<br>NO........2 → ***PLACE C***	IF I HAVE COMPANY (RELATIVES, CHILDREN)...............................1<br>IF I CAN ARRANGE MY OWN EXPENSES (FOR TRANSPORT)............2<br>IF I FOLLOW PURDAH / DRESS ACCEPTABLY...............................3<br>OTHER (SPECIFY)..4<br>UNDER NO CIRCUMSTANCES WOULD I BE ALLOWED TO GO...........5 → ***PLACE C***	YES......1<br>NO........2
**C**	Visit family or relatives				YES......1<br>NO........2 → ***PLACE D***	IF I HAVE COMPANY (RELATIVES, CHILDREN)...............................1<br>IF I CAN ARRANGE MY OWN EXPENSES (FOR TRANSPORT)............2<br>IF I FOLLOW PURDAH / DRESS ACCEPTABLY...............................3<br>OTHER (SPECIFY)..4<br>UNDER NO CIRCUMSTANCES WOULD I BE ALLOWED TO GO...........5 → ***PLACE D***	YES......1<br>NO........2
**D**	Visit a friend / neighbor's house				YES......1<br>NO........2 → ***PLACE E***	IF I HAVE COMPANY (RELATIVES, CHILDREN)...............................1<br>IF I CAN ARRANGE MY OWN EXPENSES (FOR TRANSPORT)............2<br>IF I FOLLOW PURDAH / DRESS ACCEPTABLY...............................3<br>OTHER (SPECIFY)..4<br>UNDER NO CIRCUMSTANCES WOULD I BE ALLOWED TO GO...........5 → ***PLACE E***	YES......1<br>NO........2
**E**	Hospital / clinic / doctor (seek health service)				YES......1<br>NO........2 → ***PLACE F***	IF I HAVE COMPANY (RELATIVES, CHILDREN)...............................1<br>IF I CAN ARRANGE MY OWN EXPENSES (FOR TRANSPORT)............2<br>IF I FOLLOW PURDAH / DRESS ACCEPTABLY...............................3<br>OTHER (SPECIFY)..4<br>UNDER NO CIRCUMSTANCES WOULD I BE ALLOWED TO GO...........5 → ***PLACE F***	YES......1<br>NO........2

DRAFT

		Who usually decides whether you can go to [PLACE]?<br><br>**ENTER UP TO THREE (3) MEMBER IDs**<br><br>**IF RESPONSE IS MEMBER ID (SELF) ONLY → NEXT PLACE**<br><br>**OTHER CODES:**<br>NON-HH MEMBER.......94<br>NOT APPLICABLE.......98			Does your husband/partner or other household member object to you going alone to [PLACE]?	Under what circumstances would this person NOT object to your going to [PLACE] alone?<br><br>**CIRCLE ALL APPLICABLE**	Do these objections prevent you from going alone to [PLACE]?
**PLACE**		**G6.09**			**G6.10**	**G6.11**	**G6.12**
		**ID #1**	**ID #2**	**ID #3**			
**F**	Temple / church / mosque				YES......1<br>NO........2 → ***PLACE G***	IF I HAVE COMPANY (RELATIVES, CHILDREN)................................1<br>IF I CAN ARRANGE MY OWN EXPENSES (FOR TRANSPORT)............2<br>IF I FOLLOW PURDAH / DRESS ACCEPTABLY................................3<br>OTHER (SPECIFY)..4<br>UNDER NO CIRCUMSTANCES WOULD I BE ALLOWED TO GO..........5 → ***PLACE G***	YES......1<br>NO........2
**G**	Public village gathering or community meeting				YES......1<br>NO........2 → ***PLACE H***	IF I HAVE COMPANY (RELATIVES, CHILDREN)................................1<br>IF I CAN ARRANGE MY OWN EXPENSES (FOR TRANSPORT)............2<br>IF I FOLLOW PURDAH / DRESS ACCEPTABLY................................3<br>OTHER (SPECIFY)..4<br>UNDER NO CIRCUMSTANCES WOULD I BE ALLOWED TO GO..........5 → ***PLACE H***	YES......1<br>NO........2
**H**	Training for NGO / programs				YES......1<br>NO........2 → ***PLACE I***	IF I HAVE COMPANY (RELATIVES, CHILDREN)................................1<br>IF I CAN ARRANGE MY OWN EXPENSES (FOR TRANSPORT)............2<br>IF I FOLLOW PURDAH / DRESS ACCEPTABLY................................3<br>OTHER (SPECIFY)..4<br>UNDER NO CIRCUMSTANCES WOULD I BE ALLOWED TO GO..........5 → ***PLACE I***	YES......1<br>NO........2
**I**	Outside your community or village				YES......1<br>NO........2 → ***MODULE G7***	IF I HAVE COMPANY (RELATIVES, CHILDREN)................................1<br>IF I CAN ARRANGE MY OWN EXPENSES (FOR TRANSPORT)............2<br>IF I FOLLOW PURDAH / DRESS ACCEPTABLY................................3<br>OTHER (SPECIFY)..4<br>UNDER NO CIRCUMSTANCES WOULD I BE ALLOWED TO GO..........5 → ***MODULE G7***	YES......1<br>NO........2

DRAFT

HOUSEHOLD ID						
RESPONDENT ID						

MODULE G7: INTRAHOUSEHOLD RELATIONSHIPS

Now I'd like to ask you some questions about how you feel about some of other people in your household or family group and how you think they feel about you.<br><br>**ENTER MEMBER ID FOR EACH RELATION**<br><br>**OTHER CODES:**<br>NON-HH MEMBER.......94			Do you [NAME] respect your [RELATION]?	Does your [RELATION] respect you?	Do you trust your [RELATION] to do things that are in your best interest?	When you disagree with your [RELATION], do you feel comfortable telling him/her that you disagree?	**IS [RELATION] THE OTHER RESPONDENT WITHIN THIS HOUSEHOLD?**	Is there a co-wife within your household?
**RELATION**			**G7.02**	**G7.03**	**G7.04**	**G7.05**	**G7.06**	**G7.07**
**A**	Husband / wife	**ID #**	MOST OF THE TIME...........1<br>SOMETIMES....................2<br>RARELY..........................3<br>NEVER...........................4	MOST OF THE TIME...........1<br>SOMETIMES....................2<br>RARELY..........................3<br>NEVER...........................4	MOST OF THE TIME...........1<br>SOMETIMES....................2<br>RARELY..........................3<br>NEVER...........................4	MOST OF THE TIME...........1<br>SOMETIMES....................2<br>RARELY..........................3<br>NEVER...........................4	YES......1 → ***RELATION C***<br>NO........2	
**B**	Other respondent within the household	**ID #**	MOST OF THE TIME...........1<br>SOMETIMES....................2<br>RARELY..........................3<br>NEVER...........................4	MOST OF THE TIME...........1<br>SOMETIMES....................2<br>RARELY..........................3<br>NEVER...........................4	MOST OF THE TIME...........1<br>SOMETIMES....................2<br>RARELY..........................3<br>NEVER...........................4	MOST OF THE TIME...........1<br>SOMETIMES....................2<br>RARELY..........................3<br>NEVER...........................4		
**C**	**IF RESPONDENT IS MALE:**<br>Father (or adapt this category to capture other important relationship)<br><br>**IF RESPONDENT IS FEMALE:**<br>Mother-in-law	**ID #**	MOST OF THE TIME...........1<br>SOMETIMES....................2<br>RARELY..........................3<br>NEVER...........................4	MOST OF THE TIME...........1<br>SOMETIMES....................2<br>RARELY..........................3<br>NEVER...........................4	MOST OF THE TIME...........1<br>SOMETIMES....................2<br>RARELY..........................3<br>NEVER...........................4	MOST OF THE TIME...........1<br>SOMETIMES....................2<br>RARELY..........................3<br>NEVER...........................<br>4***IF RESPONDENT IS MALE → MODULE G8(A)***		YES......1<br>NO........2 →<br>***MODULE G8(A)***
**D**	Most senior co-wife (the person who was in the household just before you, or, if you are the senior wife, the one who married into the household after you)	**ID #**	MOST OF THE TIME...........1<br>SOMETIMES....................2<br>RARELY..........................3<br>NEVER...........................<br>4	MOST OF THE TIME...........1<br>SOMETIMES....................2<br>RARELY..........................3<br>NEVER...........................<br>4	MOST OF THE TIME...........1<br>SOMETIMES....................2<br>RARELY..........................3<br>NEVER...........................<br>4	MOST OF THE TIME...........1<br>SOMETIMES....................2<br>RARELY..........................3<br>NEVER...........................<br>4		

DRAFT

HOUSEHOLD ID						
RESPONDENT ID						

MODULE G8(A): AUTONOMY IN DECISION-MAKING

DRAFT

Now I am going to read you some stories about different farmers and their situations regarding different agricultural activities. This question format is different from the rest so take your time in answering. For each I will then ask you how much you are like or not like each of these people. We would like to know if you are completely different from them, similar to them, or somewhere in between. There are no right or wrong answers to these questions.<br><br>**READ ALOUD EACH STORY, SUBSEQUENT QUESTIONs, AND RESPONSE CODES. NAMES SHOULD BE ADOPTED TO LOCAL CONTEXT AND BE MALE/FEMALE DEPENDING ON THE SEX OF THE RESPONDENT. THE ORDER OF TOPICS A-D SHOULD BE RANDOMIZED, AND WITHIN EACH TOPIC, THE ORDER OF STORIES 1-4 SHOULD BE RANDOMIZED.**			Are you like this person?<br><br>**CIRCLE ONE**	Are you completely the same or somewhat the same?<br><br>**CIRCLE ONE**	Are you completely different or somewhat different?<br><br>**CIRCLE ONE**
**STORY**			**G8.01**	**G8.02**	**G8.03**
The types of crops to grow or raise for consumption and sale in market	**A1**	*"[PERSON'S NAME] cannot grow other types of crops here for consumption and sale in market. Beans, sweet potato and maize are the only crops that grow here."*	YES...1<br>NO.....2 → ***G8.03***	COMPLETELY THE SAME....1 → ***A2***<br>SOMEWHAT THE SAME.......2 → ***A2***	COMPLETELY DIFFERENT....1<br>SOMEWHAT DIFFERENT.......2
	**A2**	*"[PERSON'S NAME] is a farmer and grows beans, sweet potato, and maize because her spouse, or another person or group in her community tells her she must grow these crops. She does what they tell her to do."*	YES...1<br>NO.....2 → ***G8.03***	COMPLETELY THE SAME....1 → ***A3***<br>SOMEWHAT THE SAME.......2 → ***A3***	COMPLETELY DIFFERENT....1<br>SOMEWHAT DIFFERENT.......2
	**A3**	*"[PERSON'S NAME] grows the crops for agricultural production that her family or community expect. She wants them to approve of her as a good farmer."*	YES...1<br>NO.....2 → ***G8.03***	COMPLETELY THE SAME....1 → ***A4***<br>SOMEWHAT THE SAME.......2 → ***A4***	COMPLETELY DIFFERENT....1<br>SOMEWHAT DIFFERENT.......2
	**A4**	*"[PERSON'S NAME] chooses the crops that she personally wants to grow for consumption and sale in market and thinks are best for herself and her family. She values growing these crops. If she changed her mind, she could act differently."*	YES...1<br>NO.....2 → ***G8.03***	COMPLETELY THE SAME....1 → ***B1***<br>SOMEWHAT THE SAME.......2 → ***B1***	COMPLETELY DIFFERENT....1<br>SOMEWHAT DIFFERENT.......2
Livestock raising	**B1**	*"[PERSON'S NAME] cannot raise any livestock other than what she has. These are all that do well here."*	YES...1<br>NO.....2 → ***G8.03***	COMPLETELY THE SAME....1 → ***B2***<br>SOMEWHAT THE SAME.......2 → ***B2***	COMPLETELY DIFFERENT....1<br>SOMEWHAT DIFFERENT.......2
	**B2**	*"[PERSON'S NAME] raises the types of livestock she does because her spouse, or another person or group in her community tell her she must use these breeds. She does what they tell her to do."*	YES...1<br>NO.....2 → ***G8.03***	COMPLETELY THE SAME....1 → ***B3***<br>SOMEWHAT THE SAME.......2 → ***B3***	COMPLETELY DIFFERENT....1<br>SOMEWHAT DIFFERENT.......2
	**B3**	*"[PERSON'S NAME] raises the kinds of livestock that her family or community expect. She wants them to approve of her as a good livestock raiser."*	YES...1<br>NO.....2 → ***G8.03***	COMPLETELY THE SAME....1 → ***B4***<br>SOMEWHAT THE SAME.......2 → ***B4***	COMPLETELY DIFFERENT....1<br>SOMEWHAT DIFFERENT.......2

	**B4**	*"[PERSON'S NAME] chooses the types of livestock that she personally wants to raise and thinks are good for herself and her family. She values raising these types. If she changed her mind, she could act differently."*	YES...1<br>NO.....2 → ***G8.03***	COMPLETELY THE SAME....1 → ***C1***<br>SOMEWHAT THE SAME.......2 → ***C1***	COMPLETELY DIFFERENT....1<br>SOMEWHAT DIFFERENT.......2

READ ALOUD EACH STORY, SUBSEQUENT QUESTIONs, AND RESPONSE CODES. NAMES SHOULD BE ADOPTED TO LOCAL CONTEXT AND BE MALE/FEMALE DEPENDING ON THE SEX OF THE RESPONDENT.			Are you like this person?<br><br>CIRCLE ONE	Are you completely the same or somewhat the same?<br><br>CIRCLE ONE	Are you completely different or somewhat different?<br><br>CIRCLE ONE
**STORY**			**G8.01**	**G8.02**	**G8.03**
Taking crops or livestock (incl. eggs or milk) to the market (or not)	**C1**	*"There is no alternative to how much or how little of her crops or livestock [PERSON'S NAME] can take to the market. She is taking the only possible amount."*	YES...1<br>NO.....2 → ***G8.03***	COMPLETELY THE SAME....1 → ***C2***<br>SOMEWHAT THE SAME.......2 → ***C2***	COMPLETELY DIFFERENT....1<br>SOMEWHAT DIFFERENT.......2
	**C2**	*"[PERSON'S NAME] takes crops and livestock to the market because her spouse, or another person or group in her community tell her she must sell them there. She does what they tell her to do."*	YES...1<br>NO.....2 → ***G8.03***	COMPLETELY THE SAME....1 → ***C3***<br>SOMEWHAT THE SAME.......2 → ***C3***	COMPLETELY DIFFERENT....1<br>SOMEWHAT DIFFERENT.......2
	**C3**	*"[PERSON'S NAME] takes the crops and livestock to the market that her family or community expect. She wants them to approve of her."*	YES...1<br>NO.....2 → ***G8.03***	COMPLETELY THE SAME....1 → ***C4***<br>SOMEWHAT THE SAME.......2 → ***C4***	COMPLETELY DIFFERENT....1<br>SOMEWHAT DIFFERENT.......2
	**C4**	*"[PERSON'S NAME] chooses to take the crops and livestock to market that she personally wants to sell there, and thinks is best for herself and her family. She values this approach to sales. If she changed her mind, she could act differently."*	YES...1<br>NO.....2 → ***G8.03***	COMPLETELY THE SAME....1 → ***D1***<br>SOMEWHAT THE SAME.......2 → ***D1***	COMPLETELY DIFFERENT....1<br>SOMEWHAT DIFFERENT.......2
How to use income generated from agricultural and non-agricultural activities	**D1**	*"There is no alternative to how [PERSON'S NAME] uses her income. How she uses her income is determined by necessity."*	YES...1<br>NO.....2 → ***G8.03***	COMPLETELY THE SAME....1 → ***D2***<br>SOMEWHAT THE SAME.......2 → ***D2***	COMPLETELY DIFFERENT....1<br>SOMEWHAT DIFFERENT.......2
	**D2**	*"[PERSON'S NAME] uses her income how her spouse, or another person or group in her community tell her she must use it there. She does what they tell her to do."*	YES...1<br>NO.....2 → ***G8.03***	COMPLETELY THE SAME....1 → ***D3***<br>SOMEWHAT THE SAME.......2 → ***D3***	COMPLETELY DIFFERENT....1<br>SOMEWHAT DIFFERENT.......2
	**D3**	*"[PERSON'S NAME] uses her income in the way that her family or community expect. She wants them to approve of her."*	YES...1<br>NO.....2 → ***G8.03***	COMPLETELY THE SAME....1 → ***D4***<br>SOMEWHAT THE SAME.......2 → ***D4***	COMPLETELY DIFFERENT....1<br>SOMEWHAT DIFFERENT.......2
	**D4**	*"[PERSON'S NAME] chooses to use her income how she personally wants to, and thinks is best for herself and her family. She values using her income in this way. If she changed her mind, she could act differently."*	YES...1<br>NO.....2 → ***G8.03***	COMPLETELY THE SAME...1→***G8.04***<br>SOMEWHAT THE SAME....2 →***G8.04***	COMPLETELY DIFFERENT....1<br>SOMEWHAT DIFFERENT.......2

DRAFT

MODULE G8(B): NEW GENERAL SELF-EFFICACY SCALE

Now I'm going to ask you some questions about different feelings you might have. Please listen to each of the following statements. Think about how each statement relates to your life, and then tell me how much you agree or disagree with the statement on a scale of 1 to 5, where 1 means you "strongly disagree" and 5 means you "strongly agree." **(Note: Randomize order of statements)**

STATEMENTS		G8.04
A	I will be able to achieve most of the goals that I have set for myself.	STRONGLY DISAGREE .... 1<br>DISAGREE .... 2<br>NEITHER AGREE NOR DISAGREE .... 3<br>AGREE .... 4<br>STRONGLY AGREE 5
B	When facing difficult tasks, I am certain that I will accomplish them.	STRONGLY DISAGREE .... 1<br>DISAGREE .... 2<br>NEITHER AGREE NOR DISAGREE .... 3<br>AGREE .... 4<br>STRONGLY AGREE 5
C	In general, I think that I can obtain outcomes that are important to me.	STRONGLY DISAGREE .... 1<br>DISAGREE .... 2<br>NEITHER AGREE NOR DISAGREE .... 3<br>AGREE .... 4<br>STRONGLY AGREE 5
D	I believe I can succeed at most any endeavor to which I set my mind	STRONGLY DISAGREE .... 1<br>DISAGREE .... 2<br>NEITHER AGREE NOR DISAGREE .... 3<br>AGREE .... 4<br>STRONGLY AGREE 5
E	I will be able to successfully overcome many challenges.	STRONGLY DISAGREE .... 1<br>DISAGREE .... 2<br>NEITHER AGREE NOR DISAGREE .... 3<br>AGREE .... 4<br>STRONGLY AGREE 5
F	I am confident that I can perform effectively on many different tasks.	STRONGLY DISAGREE .... 1<br>DISAGREE .... 2<br>NEITHER AGREE NOR DISAGREE .... 3<br>AGREE .... 4<br>STRONGLY AGREE 5
G	Compared to other people, I can do most tasks very well.	STRONGLY DISAGREE .... 1<br>DISAGREE .... 2<br>NEITHER AGREE NOR DISAGREE .... 3<br>AGREE .... 4<br>STRONGLY AGREE 5
H	Even when things are tough, I can perform quite well.	STRONGLY DISAGREE .... 1<br>DISAGREE .... 2<br>NEITHER AGREE NOR DISAGREE .... 3<br>AGREE .... 4<br>STRONGLY AGREE 5

DRAFT

MODULE G8(C): LIFE SATISFACTION

The following questions ask how satisfied you feel with your life as a whole, on a scale from 1 to 5, where 1 means you feel "very dissatisfied" and 5 means you feel "very satisfied."

	STATEMENTS	G8.05
A	Overall, how satisfied are you with life as a whole these days?	VERY DISSATISFIED ...... 1<br>DISSATISFIED ...... 2<br>NEITHER SATISFIED NOR DISSATISFIED ...... 3<br>SATISFIED ...... 4<br>VERY SATISFIED 5
B	Overall, how satisfied with your life were you 5 years ago?	VERY DISSATISFIED ...... 1<br>DISSATISFIED ...... 2<br>NEITHER SATISFIED NOR DISSATISFIED ...... 3<br>SATISFIED ...... 4<br>VERY SATISFIED 5
C	As your best guess, overall how satisfied with your life do you expect to feel 5 years from today?	VERY DISSATISFIED ...... 1<br>DISSATISFIED ...... 2<br>NEITHER SATISFIED NOR DISSATISFIED ...... 3<br>SATISFIED ...... 4<br>VERY SATISFIED 5

DRAFT

HOUSEHOLD ID						
RESPONDENT ID						

MODULE G9. Attitudes about Domestic Violence

Now I would like to ask about your opinion on the following issues. Please keep in mind that I am not asking about your personal experience or whether the following scenarios have happened to you. I would only like to know whether you think the following issues are acceptable.		In your opinion, is a husband justified in hitting or beating his wife in the following situations?
**SITUATION**		**G9.01**
**A**	If she goes out without telling him?	YES........1<br>NO........2<br>DON'T KNOW........97
**B**	If she neglects the children?	YES........1<br>NO........2<br>DON'T KNOW........97
**C**	If she argues with him?	YES........1<br>NO........2<br>DON'T KNOW........97
**D**	If she refuses to have sex with him?	YES........1<br>NO........2<br>DON'T KNOW........97
**E**	If she burns the food?	YES........1<br>NO........2<br>DON'T KNOW........97

END OF QUESTIONAIRE. FILL OUT COVER PAGE OUTCOME G1.05.

DRAFT
