# Supplementary material for: Measurement properties of the project-level Women's Empowerment in Agriculture Index
Source: World Dev. 2019 Dec;124:104639. doi: 10.1016/j.worlddev.2019.104639 (PMC6876673; doi:10.1016/j.worlddev.2019.104639)
Supplement: Supplementary data 2 [file mmc2.docx]

**Appendix 2. Comparison of pro-WEAI Indicator Definitions and Classification of Survey Items for Item-Response-Theory Analysis**

| **Indicator** | **Definition of adequacy** | **Difference compared to original WEAI** | **Comparison to Item Response Theory Analysis** |
| --- | --- | --- | --- |
| ***Intrinsic Agency*** | | | |
| Autonomy in  income | More motivated by own values than by coercion or fear of others’ disapproval: *Relative Autonomy Index^A^* score>=1  RAI score is calculated by summing responses to the three vignettes (yes=1; no=0), using the following weighting scheme: -2 for vignette 2 (external motivation), -1 for vignette 3 (introjected motivation), and +3 for vignette 4 (autonomous motivation) | Based on “Autonomy in production” indicator in the WEAI but now focuses exclusively on the use of income generated from agricultural and non-agricultural activities and uses a new vignette-based survey instrument. | *Autonomy in use of income*. Vignettes, inspired by the Relative Autonomy Index (Ryan & Deci, 2000) sought to measure motivations behind women’s actions with respect to their income, distinguishing external and internal forms of regulation. Women’s ordinal responses—completely the same (=0), somewhat the same (=1), somewhat different (=2), and completely different (=3)—to the question, ‘How similar are you to someone who…’   1. uses her income as determined by necessity 2. uses her income how her family or community tells her she must (external) 3. uses her income how her family or community expects because she wants them to approve of her (external) 4. chooses to use her income how she wants to and thinks is best for herself and her family (internal) |
| Self-efficacy | "Agree" or greater on average with self-efficacy questions: *New General Self-Efficacy Scale^B^* score>=32 | Not included in the WEAI | Not analyzed because not available in both datasets |
| Attitudes about intimate partner violence against women | Believes husband is NOT justified in hitting or beating his wife in all 5 scenarios:^C^   1. She goes out without telling him 2. She neglects the children 3. She argues with him 4. She refuses to have sex with him 5. She burns the food | Not included in the WEAI | *Intrinsic agency in the right to bodily integrity.* Women’s yes=0/no=1 responses to the question, ‘Is a husband justified in hitting his wife if…,’   1. she goes out without telling him? 2. she neglects the children? 3. she argues with him? 4. she refuses to have sex with him 5. she burns the food? |
| Respect among household members | Meets ALL of the following conditions related to another household member:   1. Respondent respects relation (MOST of the time) AND 2. Relation respects respondent (MOST of the time) AND 3. Respondent trusts relation (MOST of the time) AND 4. Respondent is comfortable disagreeing with relation (MOST of the time) | Not included in the WEAI | Not analyzed because not considered a measure of agency, by the definitions used in this manuscript |
| ***Instrumental Agency*** | | | |
| Input in productive decisions | Meets at least ONE of the following conditions for ALL of the agricultural activities they participate in   1. Makes related decision solely, 2. Makes the decision jointly and has at least some input into the decisions 3. Feels could make decision if wanted to (to at least a MEDIUM extent) | Included in the WEAI, but now uses a stricter adequacy cut-off | Analyzed separately as *intrinsic agency in livelihoods activities* and *instrumental agency in livelihoods activities*.  *Intrinsic agency in livelihoods activities* was captured using women’s responses to the question, ‘To what extent do you feel you can participate in decisions regarding [ACTIVITY] if you want(ed) to.’ Of 10 activities listed, examples were ‘raising poultry,’ ‘high-value crop farming,’ and ‘wage or salary employment.’  Response options captured participation (yes/no) and the extent that participants felt able to influence decisions about the activity (0=not at all, 1=small extent, 2=medium extent, 3=large extent). |
| Ownership of land and other assets | Owns, either solely or jointly, at least ONE of the following:   1. At least THREE small assets (poultry, nonmechanized equipment, or small consumer durables) 2. At least TWO large assets 3. Land | Included in the WEAI, but now uses a stricter adequacy cut-off | Not analyzed because too few items for adequate IRT analysis |
| Access to and decisions on financial services | Meets at least ONE of the following conditions:   1. Belongs to a household that used a source of credit in the past year AND participated in at least ONE sole or joint decision about it 2. Belongs to a household that did not use credit in the past year but could have if wanted to from at least ONE source 3. Has access, solely or jointly, to a financial account | Based on “Access to and decisions on credit” indicator in the WEAI, but now includes access to financial accounts | *Instrumental agency in borrowing from financial services*. Women’s responses to 3 questions   1. Who made the decision to borrow from [SOURCE] most of the time? 2. Who made the decision about what to do with the money from [SOURCE] most of the time? 3. Who was responsible for repaying the money borrowed from [SOURCE]?   Examples of the 6 financial services listed were specific formal lenders, informal lender, and, friends or relatives  Response options were nominal, capturing first whether the house-hold was able to borrow from each source if it wanted to (yes/no), then whether the household borrowed from this source in the past 12 months (yes/no), and if so, whether the respondent was involved in decisions about borrowing (yes/no). |
| Control over use of income | Has input in decisions related to how to use BOTH income and output from ALL of the agricultural activities they participate in AND has input in decisions related to income from ALL non-agricultural activities they participate in, unless no decision was made | Included in the WEAI, but now uses a stricter adequacy cut-off | *Instrumental agency in the sale or use of outputs* from 6 of the 10 (agricultural) livelihoods activities was captured using women’s responses to the question, ‘How much input did you have in decisions about…how much of the outputs of [ACTIVITY] to keep for consumption at home rather than selling?’  *Instrumental agency in the use of income* generated from 8 of the 10 livelihoods activities was captured using women’s responses to the question, ‘How much input did you have in decisions about…how to use income generated from [ACTIVITY].’  Response options for all 10 livelihoods activities were ‘partially ordered’ by design, in that a nominal category captured women’s ‘non-participation’ in each activity, and ordered categories captured the amount of input that participants reported having in decisions about the activity, its outputs, or income generated (0=little to no decisions, 1=some decisions, 2=most or all decisions). |
| Work balance | Works less than 10.5 hours per day:  Workload = time spent in primary activity + (1/2) time spent in childcare as a secondary activity | Similar to ‘Workload” indicator in the WEAI but restricts the measurement of secondary activities to a single activity: childcare. | Not analyzed because not a standard set of items for scale development |
| Visiting important locations | Meets at least ONE of the following conditions:   1. Visits at least TWO locations at least ONCE PER WEEK of [city, market, family/relative], or 2. Visits least ONE location at least ONCE PER MONTH of [health facility, public meeting] | Not included in the WEAI | Not analyzed because frequency of visitation is not a clear measure of instrumental agency |
| ***Collective Agency*** | | | |
| Group membership | Active member of at least ONE group | Same as in the WEAI | *Intrinsic agency in group membership*. Women’s responses to the question, ‘To what extent do you feel you can influence decisions in [GROUP]?’  Examples of eight groups listed were ‘agriculture/livestock,’ ‘credit or microfinance,’ and ‘religious.’  Response options captured the presence of a group (yes/no), active membership in the group (yes/no), and the extent that active members felt they could influence decisions in the group (0=not at all, 1=small extent, 2=medium extent, 3=high extent). |
| Membership in influential groups | Active member of at least ONE group that can influence the community to at least a MEDIUM extent | Not included in the WEAI |  |

^1^ As defined by Malapit et al. (2019)
